# Thermalization and relaxation mediated by phonon management in tin-lead perovskites

**DOI:** 10.1038/s41377-023-01236-w

**Published:** 2023-08-30

**Authors:** Linjie Dai, Junzhi Ye, Neil C. Greenham

**Affiliations:** Cavendish Laboratory, University of Cambridge, 19 J. J. Thomson Avenue, Cambridge, CB3 0HE UK

**Keywords:** Photonic devices, Electronics, photonics and device physics

## Abstract

Understanding and control of ultrafast non-equilibrium processes in semiconductors is key to making use of the full photon energy before relaxation, leading to new ways to break efficiency limits for solar energy conversion. In this work, we demonstrate the observation and modulation of slow relaxation in uniformly mixed tin-lead perovskites (MASn_x_Pb_1-x_I_3_ and CsSn_x_Pb_1-x_I_3_ nanocrystals). Transient absorption measurements reveal that slow cooling mediated by a hot phonon bottleneck effect appears at carrier densities above ~10^18^ cm^−3^ for tin-lead alloy nanocrystals, and tin addition is found to give rise to suppressed cooling. Within the alloy nanoparticles, the combination of a newly introduced high-energy band, screened Fröhlich interaction, suppressed Klemens decay and reduced thermal conductivity (acoustic phonon transport) with increased tin content contributes to the slowed relaxation. For inorganic nanocrystals where defect states couple strongly with carriers, sodium doping has been confirmed to benefit in maintaining hot carriers by decoupling them from deep defects, leading to a decreased energy-loss rate during thermalization and an enhanced hot phonon bottleneck effect. The slow cooling we observe uncovers the intrinsic photophysics of perovskite nanocrystals, with implications for photovoltaic applications where suppressed cooling could lead to hot-carrier solar cells.

## Introduction

Halide perovskites have recently gained substantial attention due to their remarkable performance in solar cells^1–4^. In addition, slow hot carrier cooling, the key process related to hot carrier solar cells breaking the Shockley-Queisser limit, was observed at high excitation densities due to the ‘hot phonon bottleneck’ effect in lead-based perovskites^5,6^ and lead-based perovskite nanocrystals^7–12^ where defect tolerance is known to make a remarkable contribution to the optoelectronic properties. The attractive optoelectronic properties of lead-halide perovskites for solar cell applications have drawn great attention to the search for alternative materials that could replicate the exceptional intrinsic photophysical properties in lead-based perovskites, but also overcome their toxicity. There has been much interest in the development of tin-based perovskites due to their lower toxicity and the potential of preserving hot carriers as tin-containing bulk perovskites have previously demonstrated^13–15^. However, for nanocrystal systems where surface states and inhomogeneous cation disorder are believed to be more important than the intrinsic semiconductor structures, to what extent the relaxation process can be manipulated by tin substitution and possible ways to overcome the defects need to be further studied.

**Fig. 1:**
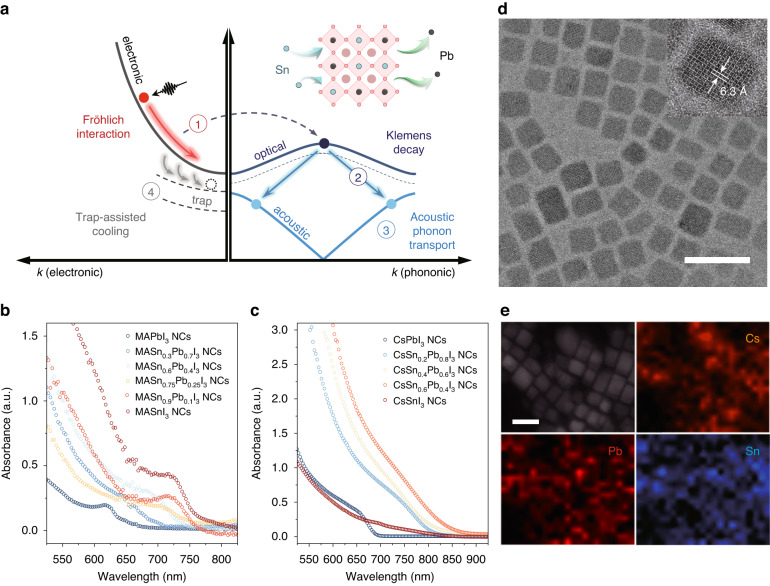
Characterization of Sn-Pb alloy perovskite NCs. **a** Schematic illustration of relaxation processes in semiconductors. UV-Vis spectra of **b** MASn_x_Pb_1-x_I_3_ NCs (~3 nm) and **c** CsSn_x_Pb_1-x_I_3_ NCs (~10 nm). **d** TEM and high-resolution TEM (insert) images of Na-passivated CsSn_0.4_Pb_0.6_I_3_ NCs synthesized at 170 °C (scale bar, 30 nm). **e** STEM image (scale bar, 20 nm) and EDS map of CsSn_0.4_Pb_0.6_Br_3_ NCs synthesized at 170 °C

In this work, we explore the hot phonon bottleneck in tin-lead perovskite nanocrystals (which is distinct from the classical phonon bottleneck effect^16^) and investigate the role of tin substitution in thermalization and relaxation processes. In general, carrier relaxation is controlled by a number of processes, as shown in Fig. 1a, including (1) the Fröhlich interaction between hot carriers and metal-halide sub-lattices, which predominantly excites high-energy longitudinal-optical (LO) phonons; (2) the decay of LO phonons to low-energy acoustic phonons; and (3) the propagation of acoustic phonons, macroscopically depicted as heat dissipation to the environment. By controlling the chemical structure (A, B-site cations) in confined nanocrystals, we are able to understand and optimize the non-equilibrium dynamics so as to maximize carrier temperature. However, nanocrystals introduce further sources of defects compared to bulk materials, particularly in tin-containing systems which are less tolerant to defects. These defects are believed to be more important in the relaxation process (path (4) in Fig. 1a). Here, we also investigate a particular strategy to decouple hot carriers from defects and thus prolong the carrier lifetime. Our results provide a detailed understanding of how electronic structure manipulation allows control over relaxation, and such promising features would make an Sn-Pb alloy nanocrystal system an ideal candidate for hot carrier solar cells.

## Results

Inorganic CsPbI_3_ NCs, CsSnI_3_ NCs, CsSn_x_Pb_1-x_I_3_ NCs, and Na-doped CsSn_x_Pb_1-x_I_3_ NCs were synthesized using a hot injection method^17–20^. MASnI_3_ NCs and MASn_x_Pb_1-x_I_3_ NCs were synthesized by a hot injection method similar to the cesium-based systems^21^. A metal halide precursor solution containing pure SnI_2_ or a mixture of SnI_2_ and PbI_2_ was injected into methylamine solution (in octadecene solvent with oleic acid and oleylamine). Due to the insufficient solubility of PbI_2_ in trioctylphosphine, additional ligands such as oleic acid can be used to dissolve PbI_2_. A ligand-assisted reprecipitation (LARP) method was used to synthesize chemically-stable MAPbI_3_ NCs^22^. The MAPbI_3_ precursor was prepared by dissolving MAI and PbI_2_ in acetonitrile (a ‘good’ solvent) while MA gas was introduced to the mixture to promote dissolution. The MAPbI_3_ NCs were synthesized by injecting the precursor into toluene (a ‘bad’ solvent) with the presence of oleic acid and oleylamine at temperatures ranging from ~25 °C to 60 °C. The immediate addition of SnI_2_-TOP stock solution after the injection of MAPbI_3_ precursor leads to the formation of MASn_x_Pb_1-x_I_3_ nanocrystals. This synthesis method gives enhanced size tunability (see Materials and Methods for details). Figure 1b, c show the steady-state absorption of as-synthesized NCs. Bulk MASn_x_Pb_1-x_I_3_ perovskites possess a narrower bandgap in intermediate Sn-Pb alloy (*x* ~ 0.8) compositions compared to both pure MAPbI_3_ and MASnI_3_ mother materials^23^. However, MASn_x_Pb_1-x_I_3_ NCs of a similar size of ~3 nm show the absence of the bandgap bowing effect (see photoluminescence of Sn-based and Sn-Pb NCs in Figs. S1 and S2). Figure 1d shows the TEM images of Na-doped CsSn_0.4_Pb_0.6_I_3_ NCs synthesized at 170 °C (see TEM images of MASn_x_Pb_1-x_I_3_ NCs and CsSn_x_Pb_1-x_I_3_ NCs in Figs. S3–S10). Figure 1e shows scanning transmission electron microscopy (STEM) images with energy-dispersive X-ray spectroscopy (EDS) maps (see STEM EDS map for Na-doped cesium tin-lead iodide NCs in Fig. S11). According to EDS elemental mapping, Cs, Pb, Sn, and Br elements share almost the same distribution pattern, consistent with the pattern formed by nanocrystals in the corresponding STEM image, indicating these elements are uniformly distributed between nanocrystals. According to the EDS line scan (Fig. S12), the Cs, Pb, Sn, and Br elements share the same trend in the change of counts per seconds. Every peak of these lines corresponds to a middle position of a nanocrystal while each minimum of these lines indicates the gap between the two neighboring nanocrystals. The results confirm that each nanocrystal is an alloy of Sn and Pb, containing similar composition. The atom percentages of Cs, Sn, Pb, and Br are ~24%, ~9%, ~13%, and ~54% respectively, corresponding to a ratio of Cs:Sn:Pb:Br ~ 6:2:3:13, broadly consistent with the perovskite chemical formula of CsSn_0.4_Pb_0.6_Br_3_.

### Hot carrier cooling in MASnI_3_ NCs

**Fig. 2:**
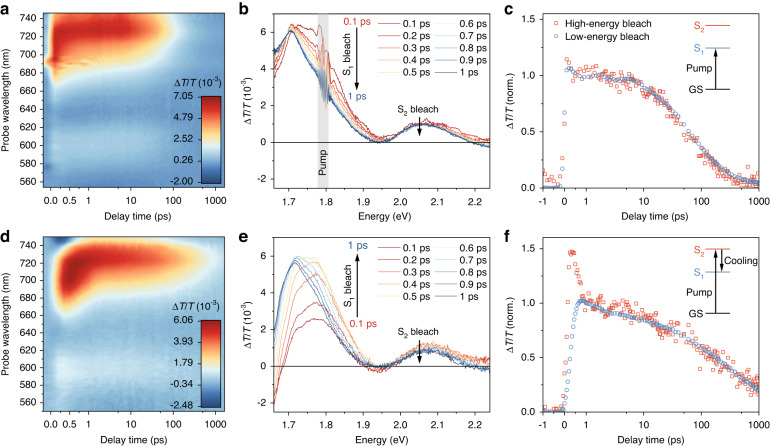
Transient absorption spectroscopy of MASnI_**3**_ NCs. **a** TA map, **b** TA spectra (0.1–1 ps), and **c** kinetics of the two bleaches of MASnI_3_ NCs under a 1.80-eV pump. **d** TA map, **e** TA spectra (0.1 ps to 1 ps), and **f** the kinetics of the two bleaches of MASnI_3_ NCs under a 3.10-eV pump

Transient absorption (TA) spectroscopy is used to reveal hot carrier dynamics in MASnI_3_ NCs after the absorption of femtosecond laser pulses. Figure 2a, d show TA maps of MASnI_3_ NCs under a lower-photon-energy pump (*ħω* = 1.80 eV, *λ* = 690 nm) and a higher-photon-energy pump (*ħω* = 3.10 eV, *λ* = 400 nm), with the same initial carrier density of 4.08 × 10^17 ^cm^−3^ (see determination of absorption cross-section, calculation of initial carrier density and estimation of the average number of absorbed photons per nanocrystal 〈*N*〉 in Supplementary Notes S1–3 and Fig. S13). Under both excitation conditions, we observe two ground state bleaches (GSB) positioned at the band edge and ~0.35 eV above the band edge, implying two excited states (S_1_ and S_2_) accessible from the ground state (GS). We attribute these two states to the dual-band electronic structure of perovskites, i.e., the existence of two conduction bands or two valence bands. A similar dual-photobleaching spectral response has been observed in lead perovskite bulk^24^ and nanocrystals^25^, and other tin-based perovskite nanocrystals^16,26^ (see TA spectra of FASnI_3_ NCs and CsSnI_3_ NCs in Fig. S14). Note that these two GSBs are the intrinsic signals of Sn^2+^ perovskites instead of the self-doping of Sn^4+^. Furthermore, in-situ TA studies confirm that an additional GSB signal (due to Sn^4+^) at an energy between the two intrinsic Sn^2+^ GSB appears during the degradation processes, which is an indicator of Sn^4+^ doping (see the change of TA spectra during degradation of MASnI_3_ NCs, FASnI_3_ NCs, and CsSnI_3_ NCs in Fig. S15). We note that there is no evidence of Sn^4+^ features in our main data for Sn^2+^-Pb^2+^ halide perovskite nanocrystals.

Figure 2b shows the TA spectra when pumping with a photon energy slightly higher than the bandgap (*ħω*_pump_ = *E*_g_ = 1.80 eV, *λ*_pump_ = 690 nm). Two bleaches decrease simultaneously with time from 0.1 to 1 ps, resulting from the repopulation of the ground state due to recombination processes. Figure 2c shows the normalized kinetics of these two bleaches. At early times, rapid rises were detected in both bleaches simultaneously, corresponding to the light absorption process (*λ*_pump_ = 690 nm, pulse width ~100 fs). Then two bleaches share extremely similar kinetics until the recovery of the ground state (~1 ns). Under a 690-nm pump, photoexcitations with energies between the high-energy band and the low-energy band are formed. As the pump does not populate the high-energy band, the kinetics are only determined by the repopulation of the ground state.

Figure 2e shows the TA spectra when pumping with photon energy significantly higher than the bandgap (*ħω*_pump_ = 3.1 eV, *λ*_pump_ = 400 nm). The high-energy bleach reaches a maximum value immediately after the femtosecond pump and then begins to decline, whereas the low-energy bleach keeps building up during this period. According to the normalized kinetics of the two bleaches (Fig. 2f), the decrease in the high-energy bleach corresponds to a concomitant increase in the low-energy bleach until the low-energy bleach peaks at ~1 ps. After carrier relaxation from the high-energy state to the low-energy state, both bleaches share almost the same dynamics. Under a 400-nm pump, hot carriers are promoted above the high-energy band. These hot carriers undergo rapid carrier-phonon scattering and cool down to the edge of the second band. The carrier relaxation from the second band (corresponding to the high-energy bleach) to the first band (corresponding to the band-edge bleach) can be directly observed from the kinetics of two bleaches. After completing this interband relaxation process (~1 ps), two bleaches share the same dynamics determined by the repopulation of the ground state.

**Fig. 3:**
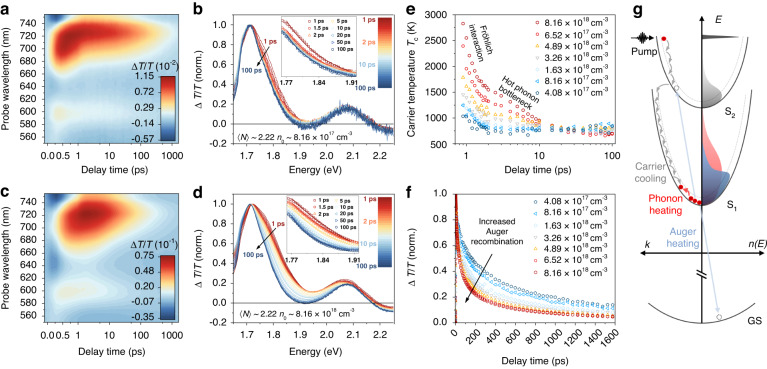
Fluence-dependent transient absorption spectroscopy of MASnI_**3**_ NCs. **a** TA map under a low-fluence 3.1-eV pump. **b** Normalized TA spectra (1–100 ps) with Boltzmann distribution fitting curves for the tail (inset). **c** TA map under a high-fluence 3.1-eV pump. **d** Normalized TA spectra (1–100 ps) with Boltzmann distribution fitting curves for the tail (inset). **e** Hot carrier temperature extracted from the TA spectra for different initial carrier densities. **f** The kinetics of the band-edge bleach under different initial carrier densities. **g** A schematic diagram of the hot carrier relaxation process after the absorption of 3.1-eV femtosecond laser pulses. The dashed lines and solid lines are the band structures before and after the bandgap renormalization. The areas filled with different colors represent the density of occupied states as a function of energy, *n*(*E*)

Hot carrier cooling was further studied by excitation-intensity-dependent TA. Figure 3a, b and c, d show the TA maps and normalized TA spectra (1–100 ps) of MASnI_3_ NCs under a low-fluence pump and a high-fluence pump with the same photon energy *ħω* = 3.1 eV. Compared with the low-fluence condition, high-fluence TA spectra show a broader high-energy tail of the band-edge bleach at early times (1 ps), and this energy tail gradually narrows over time from 1 to 100 ps, until it has almost the same spectral shape as the low-fluence case. The presence of the broadened energy tail indicates a quasi-equilibrium carrier distribution at temperatures higher than the lattice temperature. The carrier cooling process can be studied by extracting the quasi-temperature of the hot carrier distribution as a function of time (see detailed discussion in Supplementary Note S4). At early times before the complete relaxation from the second band to the first band, the carrier temperatures are not well defined as the higher-energy tail near the low-energy band edge does not include all the hot carriers. After the interband relaxation process (~1 ps), carrier temperatures are extracted by fitting the higher-energy tail with a Boltzmann distribution^6,27^ (see Fig. 3b, d insets). The higher-energy tail is chosen from ~1.77 eV to the lowest valley between two bleaches to avoid including any overlapping signals from the excitonic effect in the high-energy bleach.

Figure 3e shows the extracted carrier temperature as a function of time for different initial carrier densities, with biexponential fitting parameters listed in Table S1. For initial carrier densities less than ~10^18 ^cm^-3^, the carrier temperatures can be fit by single exponential decay, corresponding to rapid carrier cooling via the Fröhlich interaction where carrier-LO-phonon scattering has been confirmed as the dominant effect for hybrid halide perovskites at room temperature^28^. With injected carrier densities higher than ~10^18 ^cm^-3^, another slow cooling process appears after the initial sub-ps decay. This reduced relaxation rate of hot carrier temperature under high-level carrier injection results from the hot phonon bottleneck effect^6,29^, where a non-equilibrium phonon population increases the probability of carriers reabsorbing the phonon energy^9^. Apart from phonon heating, enhanced carrier-carrier interaction at high carrier concentrations could also contribute to the reduced cooling rate by a non-radiative Auger heating process^30^. Although Auger heating only plays a major role at high injected carrier densities (>10^19 ^cm^−3^)^30^, we do observe an enhanced Auger-type carrier-carrier interaction from the shortening of the decay kinetics of the band-edge bleach with increased injected carrier densities from 4.1 × 10^17 ^cm^−3^ to 8.2 × 10^18 ^cm^−3^ shown in Fig. 3f, which implies a minor contribution from Auger heating to carrier temperature.

Figure 3g shows a schematic diagram of hot carrier relaxation in MASnI_3_ perovskite nanocrystals. After the absorption of 3.1-eV femtosecond laser pulses, a non-thermal energy distribution of carriers is formed, matching the pump photon energy^10^ (see the sharp black distribution). The presence of photoexcited carriers bleaches the exciton transition because the phase-space filling leads to reduced exciton oscillator strength by decreasing the number of states contributing to the exciton. This corresponds to the two photobleaches in Fig. 3a, c. At the same time, photoexcitations promoted above the second band cause bandgaps to shrink, resulting in the renormalization (a red-shifted joint density of states) of both the first band and the second band^27^ (see band structures from dashed line to solid line), which corresponds to the early-time negative signals located at the lower-energy side of the two bleaches in Fig. 3a, c. After undergoing ultrafast carrier-carrier scattering (~100 fs), the carriers form a thermalized distribution and cool down to the edge of the high-energy band (second band) via carrier-phonon scattering (see the gray distribution). Correspondingly in Fig. 2d, the negative renormalization signal of the second band recovers along with the buildup of the high-energy bleach. These carriers then cool down through carrier-phonon interaction from the second band to the first band within 1 ps (see Fig. 2f), forming an energy tail at higher energy side of the band-edge bleach due to the state filling effect. Then, the thermodynamic equilibrium carrier distribution near the band edge can be described by a carrier temperature *T*_c_ (see the red distribution), which decreases until the carriers reach an equilibrium with the lattice temperature (see the blue distribution). The hot phonon bottleneck at high injected carrier densities contributes to the slowing of carrier relaxation, leading to prolonged average carrier cooling time of ~7 ps in MASnI_3_ NCs. The threshold for the hot phonon bottleneck effect in MASnI_3_ NCs (~10^18 ^cm^-3^) is similar to the threshold in MAPbI_3_ NCs of the same size (Fig. S16).

### Hot carrier cooling in MASn_x_Pb_1-x_I_3_ NCs

**Fig. 4:**
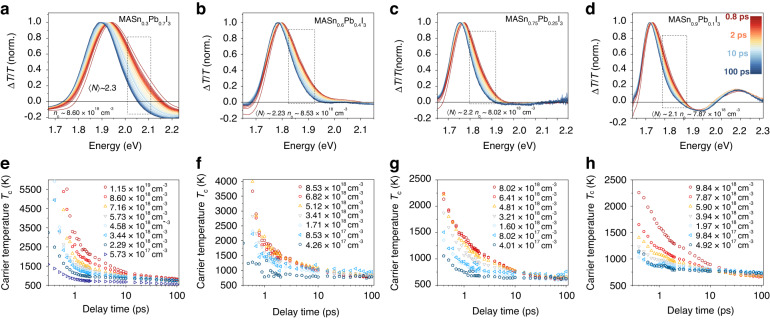
Transient absorption spectroscopy of MASn_x_Pb_1-x_I_3_ NCs. TA spectra under high injected carrier densities (0.8–100 ps) and fluence-dependent carrier temperatures of **a**, **e** MASn_0.3_Pb_0.7_I_3_ NCs, **b**, **f** MASn_0.6_Pb_0.4_I_3_ NCs, **c**, **g** MASn_0.75_Pb_0.25_I_3_ NCs, **d**, **h** MASn_0.9_Pb_0.1_I_3_ NCs

Since both MASnI_3_ and MAPbI_3_ NCs show a hot phonon bottleneck effect at high injected carrier densities, we are interested in searching for this effect in intermediate compositions. We investigate the slow cooling in MASn_x_Pb_1-x_I_3_ NCs of photoexcitations with similar excess energy as for MASnI_3_ NCs. Figure 4a–d shows the normalized spectra of MASn_x_Pb_1-x_I_3_ NCs under a high-fluence pump with similar initial carrier densities (see Fig. S17 for non-normalized spectra and TA spectra under a low-fluence pump). For all cases, a ground state bleach corresponding to the absorption band edge can be observed. The high-energy tail of the bleach signal reveals the energy distribution of hot carriers due to the state-filling effect. The hot carrier temperatures are extracted based on the TA spectra in the red dashed boxes. Figure 4e–h shows the time-dependent carrier temperature *T*_c_ under different initial carrier densities for MASn_x_Pb_1-x_I_3_ NCs. For all cases, *T*_c_ obeys a single exponential decay corresponding to the Fröhlich interaction at low injected carrier densities. When the initial carrier density reaches a threshold value of ~10^18 ^cm^−3^, a second-stage slow decay occurs, indicating the appearance of a hot phonon bottleneck.

**Fig. 5:**
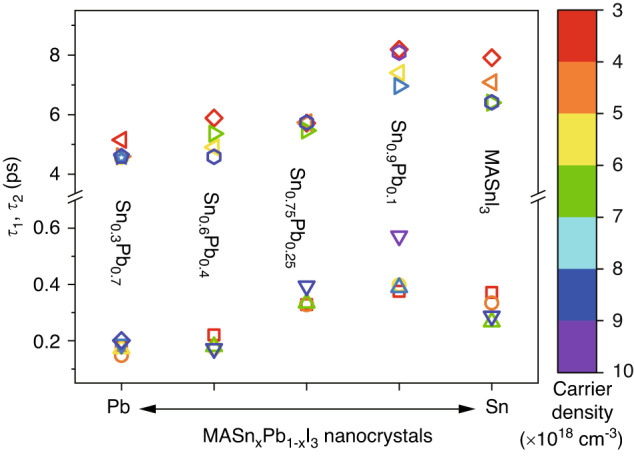
Carrier cooling time. Fitting parameters, *τ*_1_ and *τ*_2_, extracted from the biexponential decay for time-dependent carrier temperature in MASn_x_Pb_1-x_I_3_ NCs

Similar to the study of MASnI_3_ NCs, we study the slow cooling by fitting time-dependent *T*_c_ under a high-fluence pump with a bi-exponential decay. The fitting parameters (*τ*_1_ and *τ*_2_) for different initial carrier densities are plotted in Fig. 5 (see specific data points for *τ*_1_ and *τ*_2_ values in Fig. S18). For MASn_x_Pb_1-x_I_3_ NCs with a fixed Sn-to-Pb ratio, both *τ*_1_ and *τ*_2_ fluctuate only within a small range for carrier densities between 3 × 10^18 ^cm^−3^ and 1 × 10^19 ^cm^−3^, showing that within this range the cooling is only weakly dependent on pump fluence. Here *τ*_1_ corresponds to the stage of Fröhlich interaction and *τ*_2_ corresponds to a hot phonon bottleneck process.

Lead-iodide nanocrystals are known particularly for defect tolerance^31^, which leads to the slow cooling (see hot phonon bottleneck in MAPbI_3_ NCs in Fig. S16 for detail). When tin is introduced to the system, the carrier lifetime drops, associated with the increase of defect densitites. Although traps in MASn_x_Pb_1-x_I_3_ perovskite nanocrystals, where the Pb has been partially or fully replaced by Sn, dominate over intrinsic physical properties, the general trend is still preserved. In Fig. 5, for Sn-Pb alloy nanocrystals, *τ*_1_ increases as the Sn content increases, indicating a slowing of the Fröhlich interaction between carriers and polar metal-halide bonds. This results from an increase in dielectric phonon screening when introducing higher frequency Sn−I vibrational modes into the system, evidenced by the significant increase in effective dielectric constants when replacing Pb with Sn^32^. For Sn-Pb alloy nanocrystals, *τ*_2_ also increases as the Sn content increases, which corresponds to an increased hot phonon bottleneck effect. Cooling at this stage is governed by Klemens decay, where one LO phonon emitted by hot carriers decays into a pair of acoustic phonons of identical energies and opposite momenta^33^. Previous studies showed Sn perovskites have higher optical phonon frequencies, which could enlarge the energy gap between the lowest optical phonons and highest acoustic phonons^14,34^. The increase of Sn-to-Pb ratio could gradually suppress Klemens decay by opening up the phononic bandgap, leading to an increased possibility for ‘hot’ LO phonons being reabsorbed by carriers. Sn addition also reduces thermal conductivity (group velocity of acoustic phonons), where up-conversion of acoustic phonons contributes to LO phonon population^35,36^. The combination of screened Fröhlich interaction, suppressed Klemens decay, and reduced thermal conductivity (acoustic phonon transport) with increased Sn content contributes to the slowed relaxation in Sn-Pb alloy nanocrystals. When the amount of Pb is decreased to a doping level <10% (Fig. 4g), the appearance of a long-lived high-lying TA feature implies a fairly similar electronic structure between Pb-doped MASnI_3_ NCs and MASnI_3_ NCs. However, the Pb-doped NCs show a slightly longer cooling lifetime compared to pure Sn-based NCs. This is consistent with the reported results where spatial non-uniformity has been confirmed to reduce the relaxation rate due to screened polar coupling (screening of the bond polarization affecting the Fröhlich interaction)^37^. The structural distortions could also increase the chance of deformation potential interactions^15^, a secondary cooling mechanism much weaker than the Fröhlich interaction. Different fitting methods were used to test the stability and reliability of the results^38^, the detail of which is shown in Figs. S19–S21.

### Hot carrier cooling in CsSn_x_Pb_1-x_I_3_ NCs

We have so far demonstrated the presence of a hot phonon bottleneck in hybrid MASn_x_Pb_1-x_I_3_ NCs and illustrated the effects of Sn addition on suppressed relaxation, although the increasing role of defect traps severely undermines the hot carrier lifetime. For inorganic CsSn_x_Pb_1-x_Br_3_ NCs, a sub-picosecond hot carrier cooling was reported for excitation-intensity dependent measurements^39^. The short hot carrier lifetime is ascribed to strong coupling between defect traps and hot carriers which creates additional relaxation paths to compete with the intrinsic carrier-phonon interactions^39^. This also indicates NCs with an inorganic cation are more sensitive to traps compared to those with an organic cation. Here we investigate the hot carrier cooling in CsSn_x_Pb_1-x_I_3_ NCs and show that defect passivation can not only prolong the lifetime of band-edge carriers but also slow the cooling of hot carriers.

**Fig. 6:**
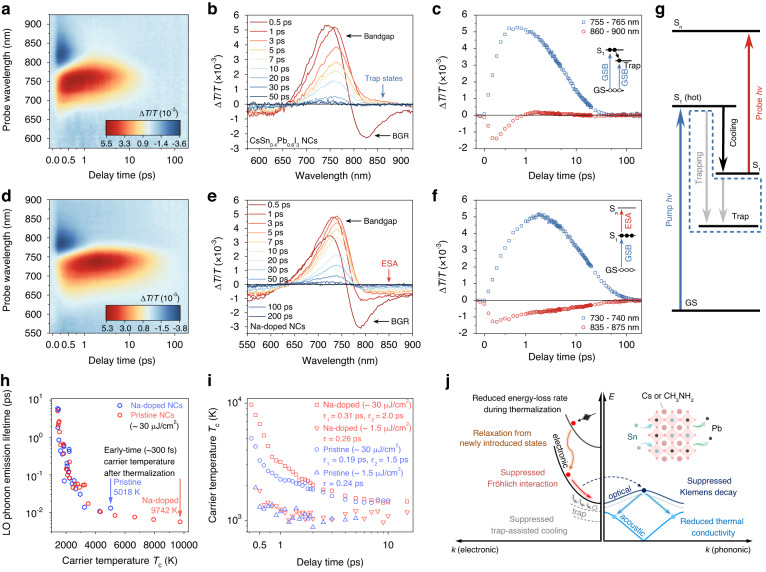
Carrier dynamics and phonon dynamics in CsSn_0.4_Pb_0.6_I_3_ and Na-doped CsSn_0.4_Pb_0.6_I_3_ NCs. **a** TA map, **b** TA spectra (0.5–50 ps), and **c** kinetics of the bleach and sub-bandgap position of CsSn_0.4_Pb_0.6_I_3_ NCs under a 2.70-eV pump (pump fluence, 4.5 μJ cm^−2^). **d** TA map, **e** TA spectra (0.5 ps to 200 ps), and **f** the kinetics of the two bleaches of Na-doped CsSn_0.4_Pb_0.6_I_3_ NCs under a 2.70-eV pump (pump fluence, 4.5 μJ cm^−2^). **g** A schematic diagram of the hot carrier relaxation process after the absorption of 2.70-eV femtosecond laser pulses. Na-doping passivates the sub-bandgap defects (see the blue dotted area) and leads to prolonged band-edge carrier lifetime and hot carrier lifetime. **h** Carrier temperature-dependent LO phonon emission lifetime in CsSn_0.4_Pb_0.6_I_3_ NCs and Na-doped CsSn_0.4_Pb_0.6_I_3_ NCs. **i** extracted carrier temperature of CsSn_0.4_Pb_0.6_I_3_ NCs and Na-doped CsSn_0.4_Pb_0.6_I_3_ NCs. **j** Carrier dynamics and phonon dynamics in Sn-Pb perovskite nanocrystals

Figure 6a–c shows the TA map, spectra, and kinetics of CsSn_0.4_Pb_0.6_I_3_ NCs, where a ground state bleach peaked at ~760 nm corresponds to the transition at bandgap energy (labeled as S_1_). The kinetics in Fig. 6c (blue dots) shows that even when partially replacing Pb with Sn, the charge carrier recombination rate increases dramatically (see kinetics of the band-edge bleach for NCs with different Sn-Pb ratio in Fig. S22). This is consistent with the previous studies where the defect chemistry of Sn-Pb alloy perovskites is considered as the dominant factor in their optoelectronic properties^40^. The negative TA signal peaked at ~825 nm before 1 ps results from bandgap renormalization. After 1 ps, a broad sub-bandgap bleach from ~825 nm to ~900 nm can be observed, indicating fast trapping of charge carriers by band-tail defect states^41–43^.

Previous work showed that sodium doping can be used to dramatically increase the photoluminescence of CsSn_x_Pb_1-x_I_3_ NCs^20^. We therefore used this strategy to reduce the number of traps in our Sn-Pb nanocrystals and prepared samples according to their method where a small amount of Na-salt was added to the precursor. Figure 6d–f shows the TA map, spectra, and kinetics of Na-doped (~0.5%) CsSn_0.4_Pb_0.6_I_3_ NCs under the same excitation condition as for undoped NCs. Similarly, a ground state bleach at ~740 nm (S_1_) with bandgap renormalization was observed. The GSB signal of Na-doped NCs exhibits decreased decay rate and weaker redshift compared to undoped NCs, indicating a prolonged carrier lifetime at the band edge due to dramatically reduced density of sub-bandgap traps. The substantial decrease in defect states allows for more intrinsic photophysics of Sn-Pb alloy perovskite system to be revealed. Different from undoped NCs, TA spectra of Na-doped NCs show long-lived photoinduced absorption (PIA) signal at the lower-energy side of the band-edge ground state bleach, and it shares the same kinetics as the S_1_ ground state bleach after the ultrafast cooling due to Fröhlich interaction (see Fig. S23). We attribute this PIA to the excited-state absorption (ESA) from the first excited state, S_1_, to higher-energy states, S_n_. After Na-doping, the sub-bandgap defect states are passivated effectively, leading to increased band-edge carrier lifetime. The absence of the low-energy bleach shoulder (corresponding to traps) also allows the intrinsic photophysics of ESA to be revealed. Careful examination of the TA signals using a higher-photon-energy pump (400 nm, 3.1 eV) and a lower-photon-energy pump (560 nm, 2.2 eV) further confirms that the removal of sub-bandgap traps leads to the observation of an ESA signal independent of pump photon energy (see Fig. S24). Figure 6g shows a schematic diagram of carrier dynamics in pristine Sn-Pb alloy NCs and Na-doped NCs as discussed above.

Figures S25a, c and S26a, b show the TA maps and TA spectra of CsSn_0.4_Pb_0.6_I_3_ NCs under a low-fluence pump and a high-fluence 460-nm pump. The spectra under a high-fluence pump show a broader high-energy tail at early times (<5 ps) than under a low-fluence pump, indicating a larger fraction of hot carriers under a high-fluence pump. The time-dependent hot carrier temperature is extracted by fitting the high-energy tail with a Boltzmann distribution, shown in Figs. S6i and S26c. The decay of carrier temperature under a low-fluence pump obeys single-exponential decay (*τ* ~ 0.24 ps), corresponding to the ultrafast Fröhlich interaction. In contrast, the decay of carrier temperature under a high-fluence pump shows bi-exponential dynamics, corresponding to a sub-picosecond Fröhlich interaction (*τ*_1_ ~ 0.2 ps) followed by a second-stage slow cooling (*τ*_2_ ~ 1.5 ps). Although the cooling at the second stage is much weaker compared to MA-based NCs, this excitation-dependent cooling indicates a hot phonon bottleneck in CsSn_x_Pb_1-x_I_3_ NCs, in contrast to the sub-picosecond cooling in the inorganic Sn-Pb nanocrystal system reported previously^39^.

The TA measurements for Na-doped NCs were performed under exactly the same conditions (pump photon energy and pump intensity) as for undoped NCs. Figures S25b, d and S26d, e show TA spectra of Na-doped CsSn_0.4_Pb_0.6_I_3_ NCs under a low-fluence pump and a high-fluence pump, respectively. Similar to undoped NCs, the high-energy tails of spectra under the high-fluence pump are broader than tails under the low-fluence pump, indicating a hot phonon bottleneck effect. The hot carrier temperature, shown in Figs. S6i and S26f, obeys a single-exponential decay (*τ*~0.26 ps) under the low-fluence pump, very similar to the case of undoped NCs. This results from the intrinsic carrier-phonon interaction induced by the same phonon mode of the CsSn_0.4_Pb_0.6_I_3_ NC matrix. Under a high-fluence pump a bi-exponential decay of carrier temperature is observed, corresponding to a sub-picosecond Fröhlich interaction (*τ*_1_ ~ 0.3 ps) followed by a second-stage slow cooling (*τ*_2_ ~ 2.0 ps) mediated by a hot phonon bottleneck whose timescale is one-third greater than the value for undoped NCs.

We further study the cooling by calculating the average carrier-LO phonon scattering time *τ*_LO_ using the following equation^44–46^:

$$-\frac{3{k}_{{\rm{B}}}}{2}\frac{{\rm{d}}{T}_{{\rm{c}}}}{{\rm{d}}t}=\frac{{\hbar}{\omega }_{{\rm{LO}}}}{{\tau }_{{\rm{LO}}}}\left[{\exp}\left(-\frac{{\hbar}{\omega }_{{\rm{LO}}}}{{k}_{{\rm{B}}}{T}_{{\rm{c}}}}\right)-{\exp}\left(-\frac{{\hbar}{\omega }_{{\rm{LO}}}}{{k}_{{\rm{B}}}{T}_{{\rm{L}}}}\right)\right]$$

where *T*_c_ is the carrier temperature, *T*_L_ is the lattice temperature, and *ω*_LO_ is the LO phonon energy for CsSn_0.4_Pb_0.6_I_3_ NCs (*ω*_LO_ ≈ 23 meV)^47,48^. Figure 6h shows the LO phonon emission lifetime in terms of carrier temperature under the same excitation conditions of 30 μJ cm^−2^ (460-nm pump). The perfect overlap of the two curves indicates the same nature of carrier-LO phonon interaction. However, the early-time carrier temperature (300 fs) of Na-doped NCs is higher than in the undoped pristine NCs under the same pump photon energy of 2.70 eV and fluence of ~30 μJ/cm^2^. We attribute this to a decreased coupling between defects and hot carriers, which blocks relaxation pathways through defects in the first few hundred femtoseconds, leading to a higher early-time carrier temperature. The dissipation of photoexcitation energy consists of several processes (Fig. 6j). Monoenergetic pulsed excitation produces an initial distribution of electrons (or holes) which is not in thermodynamic equilibrium as the energy of photogenerated carriers matches that of the absorbed photon (note that the electrons (or holes) may not be perfectly monoenergetic due to possible multiplicities of electron (or hole) states available for optical transitions). Thermalization, the first step towards establishing equilibrium, redistributes the electron (or hole) energies through carrier-carrier scattering and results in a Boltzmann distribution of electrons (or holes) with an equilibrium carrier temperature higher than the lattice temperature. This happens on a timescale of tens to hundreds of femtoseconds (<300 fs). Subsequently (>300 fs), relaxation of hot carriers takes place via the Fröhlich interaction where carriers interact inelastically with LO phonons where energy and momentum are conserved between the electron and emitted phonon. This brings the carriers and lattice to thermodynamic equilibrium. Our results show that the defect states contribute to energy loss in the thermalization process, leading to decreased carrier temperature after thermalization. Defect passivation not only prolongs the band-edge carrier lifetime but also contributes to maintaining hot carriers by decoupling hot carriers from defect states in the thermalization process.

The reduced energy loss rate during thermalization, screened Fröhlich interaction, suppressed Klemens decay and reduced thermal conductivity could contribute to the realization of hot carrier solar cells, which absorb a wide range of photon energies and extract hot carriers before they relax to the band edges. The slowing of carrier relaxation may allow hot carriers to be extracted over a narrow range of elevated (hot) energies using energy selective contacts, and the carrier-carrier interaction allows efficient renormalization of carrier energy, potentially leading to higher voltages from the cell and hence higher efficiency.

## Discussion

In summary, we demonstrate the observation and modulation of a hot phonon bottleneck effect in hybrid methylammonium tin-lead alloy halide nanocrystals (MASn_x_Pb_1-x_I_3_ NCs) and inorganic cesium tin-lead alloy halide nanocrystals (CsSn_x_Pb_1-x_I_3_ NCs). For pure tin-based MASnI_3_ nanocrystals, the carrier cooling from a second band is studied, and a hot phonon bottleneck effect is observed. Fluence-dependent measurements on MASn_x_Pb_1-x_I_3_ nanocrystals reveal that slow cooling mediated by a hot phonon bottleneck effect appears at carrier densities of ~10^18 ^cm^−3^. Within the alloy nanoparticles with random mixing of tin and lead, the combination of newly introduced high-energy band, screened Fröhlich interaction, suppressed Klemens decay and reduced thermal conductivity (acoustic phonon transport) with increased tin content contributes to the slowed relaxation. For CsSn_x_Pb_1-x_I_3_ nanocrystals where defect states couple strongly with carriers, a weak hot phonon bottleneck effect is observed. Sodium doping is used to passivate the sub-bandgap defect states in these NCs, confirmed by the excited state absorption signal from transient absorption spectroscopy. By decoupling hot carriers from defect states, decreased energy-loss rate during thermalization and enhanced hot phonon bottleneck effect are achieved in sodium-doped NCs. These results provide essential insights into the underlying mechanisms of hot carrier relaxation in hybrid and inorganic tin-lead alloy perovskite nanocrystal systems and have potential for hot carrier solar cells.

## Materials and methods

Please see the Materials and Methods section in the Supporting Information for details.

All chemicals were purchased from Sigma-Aldrich.

### Synthesis of MASn_x_Pb_1-x_I_3_ perovskite nanocrystals (hot injection method)

The mixture of dried 1-octadecene (ODE, 5 mL), dried oleic acid (OA, 160 μL), and dried oleylamine (OLA, 160 μL) were heated at 60 °C under nitrogen with vigorous stirring for 10 min. Then 172 μL of methylamine solution was added into the solution, followed by the injection of Pb-Sn precursor prepared by pre-heating the mixture of SnI_2_-stock solution and PbI_2_-stock solution to 60 °C (MASn_0.6_Pb_0.4_I_3_ NCs, SnI_2_-stock, 0.8 mL, PbI_2_-stock, 0.5 mL; MASn_0.75_Pb_0.25_I_3_ NCs, SnI_2_-stock, 0.87 mL, PbI_2_-stock, 0.33 mL; MASn_0.9_Pb_0.1_I_3_ NCs, SnI_2_-stock, 0.93 mL, PbI_2_-stock, 0.16 mL) or prepared by heating the mixture of SnI_2_ and PbI_2_ (in TOP) at 90 °C for 5 h. The reaction was kept at 60 °C for 60 s, followed by an ice-water bath to cool down to room temperature. The solution was centrifuged at 12,000 RPM for 5 min. After centrifugation, the supernatant solution was discarded and the precipitate was redispersed in hexane. The solution was centrifuged at 5000 r.p.m. for 5 min to remove aggregated nanocrystals, resulting in the supernatant of long-term colloidally stable solution.

### Synthesis of MASn_x_Pb_1-x_I_3_ perovskite nanocrystals (LARP method)

The mixture of anhydrous toluene (5 mL), oleic acid (1 mL), and oleylamine (0.2 mL) were heated to the desired temperature (between room temperature and 60 °C). The MAPbI_3_-ACN precursor (200 μL) was then injected into the solution under vigorous stirring, immediately followed by the injection of SnI_2_-TOP stock solution. The addition of 100 μL SnI_2_-TOP stock solution leads to nanocrystals with Sn:Pb ~ 1:1. The solution could be left stirring for up to 120 s for full growth of the desired nanocrystal size. The solution was centrifuged at 12,000 RPM for 5 min. After centrifugation, the supernatant solution was discarded, and the precipitate was redispersed in hexane. The solution was centrifuged at 5000 r.p.m. for 5 min to remove aggregated nanocrystals, resulting in the supernatant of a long-term colloidally stable solution.

### Synthesis of CsSn_0.6_Pb_0.4_Br_3_ perovskite nanocrystals

Cs_2_CO_3_ (0.26 g, 0.8 mmol), OA (0.8 mL), OLA (0.8 mL), and ODE (24 mL) were loaded into a three-neck flask and degassed under vacuum (~1.3 × 10^−2^ mbar) for 2 h at 110 °C. Afterwards the mixture was heated to 150 °C under N_2_ until Cs_2_CO_3_ fully dissolved. Then the Cs-precursor was heated to the reaction temperature (typically 170 °C) before the injection of SnBr_2_-PbBr_2_ precursor (11 mL) which was made by dissolving PbBr_2_ (0.8 mmol, 293.6 mg) and SnBr_2_ (3 mmol, 835.5 mg) into TOP (10 mL) and OA (1 mL) at 50 °C. The reaction vessel was kept at the injection temperature for 30 s before immersion in an ice-cold water bath. The purification was performed in an argon-filled glovebox. Nanocrystals were purified by an addition of an equal volume of 1-butanol followed by centrifugation at 8000 r.p.m. for 5 min. After centrifugation, the supernatant solution was discarded, and the precipitate was redispersed in hexane. The solution was centrifuged at 5000 r.p.m. for 5 min to remove aggregated nanocrystals, resulting in the supernatant of long-term colloidally stable solution.

### Synthesis of CsSn_x_Pb_1-x_I_3_ perovskite nanocrystals

Cs_2_CO_3_ (0.26 g, 0.8 mmol), OA (0.8 mL), OLA (0.8 mL), and ODE (24 mL) were loaded into a three-neck flask and degassed under vacuum (~1.3 × 10^−2^ mbar) for 2 h at 110 °C. Afterwards the mixture was heated to 150 °C under N_2_ until Cs_2_CO_3_ fully dissolved. Then the Cs-precursor was heated to the reaction temperature (typically 170 °C) before the injection of SnI_2_-PbI_2_-TOP precursor (5 mL) which was prepared by heating the mixture of SnI_2_ (4 mmol) and PbI_2_ (2 mmol, 1.6 mmol, 1 mmol for CsSn_0.2_Pb_0.8_I_3_ NCs, CsSn_0.4_Pb_0.6_I_3_ NCs, and CsSn_0.6_Pb_0.4_I_3_ NCs, respectively) in TOP (5 mL) at 90 °C for 5 h. The reaction vessel was kept at the injection temperature for 30 s before immersion in an ice-cold water bath. The purification was performed in an argon-filled glovebox. Nanocrystals were purified by addition of an equal volume of methyl acetate followed by centrifugation at 8000 r.p.m. for 5 min. After centrifugation, the supernatant solution was discarded and the precipitate was redispersed in hexane. The solution was centrifuged at 5000 r.p.m. for 5 min to remove aggregated nanocrystals, resulting in the supernatant of long-term colloidally stable solution.

### Transient absorptionspectroscopy measurements

Two transient absorption spectroscopy systems were used for the measurements. For TA measurements with 500–800 nm continuous probe region, the output of a Ti:sapphire amplifier system (Spectra Physics Solstice Ace) operating at 1 kHz and generating ~100-fs pulses was split into the pump and probe beam paths. The 400-nm pump pulses were created by sending the 800-nm fundamental beam of the Solstice Ace through a second harmonic generating (SHG) beta barium borate (BBO) crystal of 1-mm thickness (Eksma Optics). Wavelength tunable pump pulses (e.g., 690-nm pump) were generated in a home-built noncollinear optical parametric amplifier (NOPA). The pump was blocked by a chopper wheel rotating at 500 Hz while a computer operated a mechanical delay stage (Thorlabs DDS300-E/M) to adjust the delay between the pump and the probe. The visible broadband beam (520–780 nm) was generated in a home-built noncollinear optical parametric amplifier (NOPA), and the white light was split into two identical beams (probe and reference) by a 50/50 beamsplitter. The reference beam passing through the sample did not interact with the pump, which allows for correcting for any shot-to-shot fluctuations in the probe that would otherwise greatly increase the structured noise in the experiments. Based on this arrangement, small signals with Δ*T/T*~10^−5^ could be measured. The transmitted probe and reference pulses were collected with a dual-line array detector driven and read out by a custom-built board (Stresing Entwicklungsbüro). For TA measurements with 500–950 nm continuous probe region, a Yb amplifier (PHAROS, Light Conversion) operating at 38 kHz and generating ~200-fs pulses centered at 1030 nm with an output of 14.5 W was used. The ~200 fs pump pulse was provided by a TOPAS OPA. The white light supercontinuum probe was generated by sending in a small portion of the 1030-nm fundamental to a YAG crystal (4 mm). The transmitted probe was imaged using a Si photodiode array (Stresing S11490).

### Supplementary information

SI

## Data Availability

The data underlying this paper are available at 10.17863/CAM.100097. For the purpose of open access, the author has applied a Creative Commons Attribution (CC BY) licence to any Author Accepted Manuscript version arising from this submission.
