# Supplementary material for: Thermalization and relaxation mediated by phonon management in tin-lead perovskites
Source: Light Sci Appl. 2023 Aug 30;12:208. doi: 10.1038/s41377-023-01236-w (PMC10468496; doi:10.1038/s41377-023-01236-w)
Supplement: Supplementary file 1 — SI [file 41377_2023_1236_MOESM1_ESM.pdf]

Thermalization and relaxation mediated by phonon management in tin-lead perovskites

Linjie Dai, Junzhi Ye, and Neil C. Greenham*

Cavendish Laboratory, University of Cambridge, 19 J. J. Thomson Avenue, Cambridge CB3 0HE, UK
E-mail: ncg11@cam.ac.uk

Table of Contents

Materials and methods -- Page 4 to Page 8

Degassing of solvents and ligands (Page 4)
Preparation of the SnI_2–stock solution (Page 4)
Preparation of the PbI_2–stock solution (Page 4)
Synthesis of $MASnI_3$ perovskite nanocrystals (hot injection method) (Page 4)
Synthesis of $MASn_xPb_{1-x}I_3$ perovskite nanocrystals (hot injection method) (Page 4)
Preparation of the $MAPbI_3$-ACN precursor (Page 5)
Synthesis of $MAPbI_3$ perovskite nanocrystals (LARP method) (Page 5)
Synthesis of $MASn_xPb_{1-x}I_3$ perovskite nanocrystals (LARP method) (Page 5)
Synthesis of $CsSn_{0.6}Pb_{0.4}Br_3$ perovskite nanocrystals (Page 6)
Synthesis of $CsSn_xPb_{1-x}I_3$ perovskite nanocrystals (Page 6)
Synthesis of Na-doped $CsSn_xPb_{1-x}I_3$ perovskite nanocrystals (Page 6)
Transmission electron microscopy (TEM) measurements (Page 7)
Ultraviolet–visible (UV–Vis) spectroscopy (Page 7)
Photoluminescence (PL) and PL quantum efficiency (PLQE) measurements (Page 7)
Transient absorption (TA) spectroscopy measurements (Ti:sapphire laser system) (Page 7)
Transient absorption (TA) spectroscopy measurements (Yb:KGW laser system) (Page 8)
Time-correlated single photon counting (TCSPC) measurements (Page 8)

Supplementary notes and figures --- Page 9 to Page 39

Note 1 Calculation of pump fluence (Page 9)
Note 2 Determination of absorption cross-section of nanocrystals (Page 9)
Note 3 Calculation of the initial carrier density (Page 9)
Note 4 Discussion on models used to determine charge-carrier temperature (Page 10)

Figure S1. Photoluminescence and TCSPC of Sn-based nanocrystals (Page 11)
Figure S2. Steady-state photoluminescence of $MASn_xPb_{1-x}I_3$ nanocrystals (Page 12)
Figure S3. TEM of $MAPbI_3$ NCs synthesized at 25 °C using LARP method (Page 13)
Figure S4. TEM of $MASn_xPb_{1-x}I_3$ NCs synthesized at 25 °C using LARP method (Page 14)
Figure S5. TEM of $MAPbI_3$ NCs synthesized at 60 °C using LARP method (Page 15)
Figure S6. TEM of $MASn_xPb_{1-x}I_3$ NCs synthesized at 60 °C using LARP method (Page 16)
Figure S7. TEM of $CsSn_xPb_{1-x}I_3$ NCs synthesized at 170 °C (Page 17)
Figure S8. TEM of Na-doped $CsSn_xPb_{1-x}I_3$ NCs synthesized at 170 °C (Page 18)
Figure S9. TEM of Na-doped $CsSn_xPb_{1-x}I_3$ NCs with a large field of view (Page 19)
Figure S10. TEM of $CsSn_{0.4}Pb_{0.6}Br_3$ NCs synthesized at 170 °C (Page 20)
Figure S11. STEM image and EDS mapping of Na-doped $CsSn_xPb_{1-x}I_3$ NCs (Page 21)
Figure S12. EDS line scan of $CsSn_{0.4}Pb_{0.6}Br_3$ NCs synthesized at 170 °C (Page 22)

Figure S13. Determination of the absorption cross-section of nanocrystals (Page 23)
Figure S14. TA spectra of $FASnI_3$ NCs and $CsSnI_3$ NCs (Page 24)
Figure S15. Degradation processes of $MASnI_3$ NCs, $FASnI_3$ NCs and $CsSnI_3$ NCs (Page 25)
Figure S16. TA of $MAPbI_3$ NCs (~3 nm) under different pump fluences (Page 26)
Figure S17. Non-normalized TA spectra of MA-based Sn-Pb alloy NCs (Page 27)
Figure S18. Fitting parameters (τ_1, τ_2) of hot carrier temperature in $MASn_xPb_{1-x}I_3$ NCs (Page 28)
Figure S19. Two methods for choosing ROI for Boltzmann distribution fitting (Page 29)
Figure S20. TA spectra of $MASn_{0.6}Pb_{0.4}I_3$ NCs with ROIs according to Method 2 (Page 31)
Figure S21. Fitting parameters (τ_1, τ_2) of hot carrier temperature according to Method 2 (Page 32)
Figure S22. TA spectra and kinetics of $CsSn_xPb_{1-x}I_3$ NCs (Page 34)
Figure S23. TA kinetics of the GSB and PIA signals of Na-doped $CsSn_{0.4}Pb_{0.6}I_3$ NCs (Page 35)
Figure S24. TA of pristine and doped NCs under 400-nm and 560-nm pump (Page 36)
Figure S25. TA maps of $CsSn_{0.4}Pb_{0.6}I_3$ NCs and Na-doped $CsSn_{0.4}Pb_{0.6}I_3$ NCs (Page 37)
Figure S26. Hot phonon bottleneck in $CsSn_{0.4}Pb_{0.6}I_3$ NCs and Na-doped NCs (Page 38)
Table S1. Fitting parameters for time-dependent carrier temperture in $MASnI_3$ NCs (Page 39)

References -- Page 40

Materials and Methods

Degassing of solvents and ligands

The solutions of 1-octadecene (ODE, Sigma-Aldrich, 90%), oleic acid (OA, Sigma-Aldrich, 90%), and oleylamine (OLA, Sigma-Aldrich, 70%) were degassed separately under vacuum at 110 °C for 2 hours to produce dried 1-octadecene, dried oleic acid, and dried oleylamine. These solutions were stored in a nitrogen-filled glovebox at room temperature.

Preparation of the SnI_2–stock solution

The mixture of SnI_2 (1862.5 mg, 5 mmol, Sigma-Aldrich, beads, 99.99%) and tri-*n*-octylphosphine (TOP, 5 mL, Sigma-Aldrich, 97%) was vigorously stirred overnight at room temperature to prepare SnI_2-TOP stock solution (1 M of SnI_2).

Preparation of the PbI_2–stock solution

The mixture of PbI_2 (1152.5 mg, 10 mmol, Sigma-Aldrich, beads, 99.999%), dried oleylamine (OLA, 0.7 mL), dried oleic acid (OA, 0.7 mL) and tri-*n*-octylphosphine (TOP) was vigorously stirred overnight at 100 °C to prepare PbI_2-TOP stock solution (~0.4 M of PbI_2).

Synthesis of $MASnI_3$ perovskite nanocrystals (hot injection method)

The mixture of dried 1-octadecene (ODE, 5 mL), dried oleic acid (OA, 160 μL), and dried oleylamine (OLA, 160 μL) were heated at 60 °C under nitrogen with vigorous stirring for 10 minutes. Then 172 μL of methylamine solution (CH_3NH_2, 2.0 M in THF, Sigma-Aldrich) was added into the solution, followed by the injection of SnI_2-stock solution (1 mL). The reaction was kept at 60 °C for 60 seconds, followed by an ice-water bath to cool down to room temperature. The solution was centrifuged at 12000 RPM for 5 minutes. After centrifugation, the supernatant solution was discarded and the precipitate was redispersed in hexane. The solution was centrifuged at 5000 RPM for 5 minutes to remove aggregated nanocrystals, resulting in the supernatant of long-term colloidally stable solution.

Synthesis of $MASn_xPb_{1-x}I_3$ perovskite nanocrystals (hot injection method)

The mixture of dried 1-octadecene (ODE, 5 mL), dried oleic acid (OA, 160 μL), and dried oleylamine (OLA, 160 μL) were heated at 60 °C under nitrogen with vigorous stirring for 10 minutes. Then 172 μL of methylamine solution was added into the solution, followed by the injection of Pb-Sn precursor prepared by pre-heating the mixture of SnI_2-stock solution and PbI_2-stock solution to 60 °C ($MASn_{0.6}Pb_{0.4}I_3$ NCs, SnI_2-stock, 0.8 mL, PbI_2-stock, 0.5 mL; $MASn_{0.75}Pb_{0.25}I_3$ NCs, SnI_2-stock, 0.87 mL,

PbI_2-stock, 0.33 mL; $MASn_{0.9}Pb_{0.1}I_3$ NCs, SnI_2-stock, 0.93 mL, PbI_2-stock, 0.16 mL) or prepared by heating the mixture of SnI_2 and PbI_2 (in TOP) at 90 °C for 5 hours. The reaction was kept at 60 °C for 60 seconds, followed by an ice-water bath to cool down to room temperature. The solution was centrifuged at 12000 RPM for 5 minutes. After centrifugation, the supernatant solution was discarded and the precipitate was redispersed in hexane. The solution was centrifuged at 5000 RPM for 5 minutes to remove aggregated nanocrystals, resulting in the supernatant of long-term colloidally stable solution.

Preparation of the $MAPbI_3$-ACN precursor

MAI (2.5 mmol, 397.4 mg) and PbI_2 (2.5 mmol, 1152.5 mg) were added into anhydrous acetonitrile (ACN, 5 mL) under vigorous stirring. A nitrogen gas flow was used to degas the methylamine solution to generate methylamine gas. The methylamine gas was then bubbled through the MAI-PbI_2-ACN solution (with black precipitate) to help dissolve PbI_2, until a transparent yellow solution was formed.

Synthesis of $MAPbI_3$ perovskite nanocrystals (LARP method)

The mixture of anhydrous toluene (5 mL), oleic acid (1 mL) and oleylamine (0.2 mL) were heated to the desired temperature (from room temperature to 60 °C). The $MAPbI_3$-ACN precursor (200 μL) was then injected into the solution under vigorous stirring. The solution could be left stirring for up to 120 s for full growth of the desired nanocrystal size (e.g., $MAPbI_3$ nanocubes, 60 °C, 30 s). The solution was centrifuged at 12000 RPM for 5 minutes. After centrifugation, the supernatant solution was discarded and the precipitate was redispersed in hexane. The solution was centrifuged at 5000 RPM for 5 minutes to remove aggregated nanocrystals, resulting in the supernatant of long-term colloidally stable solution.

Synthesis of $MASn_xPb_{1-x}I_3$ perovskite nanocrystals (LARP method)

The mixture of anhydrous toluene (5 mL), oleic acid (1 mL) and oleylamine (0.2 mL) were heated to the desired temperature (between room temperature and 60 °C). The $MAPbI_3$-ACN precursor (200 μL) was then injected into the solution under vigorous stirring, immediately followed by the injection of SnI_2-TOP stock solution. The addition of 100 μL SnI_2-TOP stock solution leads to nanocrystals with Sn:Pb ~ 1:1. The solution could be left stirring for up to 120 s for full growth of the desired nanocrystal size. The solution was centrifuged at 12000 RPM for 5 minutes. After centrifugation, the supernatant solution was discarded, and the precipitate was redispersed in hexane. The solution was centrifuged at 5000 RPM for 5 minutes to remove aggregated nanocrystals, resulting in the supernatant of long-term colloidally stable solution.

Synthesis of $CsSn_{0.6}Pb_{0.4}Br_3$ perovskite nanocrystals

Cs_2CO_3 (0.26 g, 0.8 mmol), OA (0.8 mL), OLA (0.8 mL) and ODE (24 mL) were loaded into a three-neck flask and degassed under vacuum (~ 1.3 × 10^{-2} mbar) for 2 hours at 110 °C. Afterwards the mixture was heated to 150 °C under N_2 until Cs_2CO_3 fully dissolved. Then the Cs-precursor was heated to the reaction temperature (typically 170 °C) before the injection of $SnBr_2$-$PbBr_2$ precursor (11 mL) which was made by dissolving $PbBr_2$ (0.8 mmol, 293.6 mg) and $SnBr_2$ (3 mmol, 835.5 mg) into TOP (10 mL) and OA (1 mL) at 50 °C. The reaction vessel was kept at the injection temperature for 30 s before immersion in an ice-cold water bath. The purification was performed in an argon-filled glovebox. Nanocrystals were purified by an addition of an equal volume of 1-butanol followed by centrifugation at 8000 RPM for 5 minutes. After centrifugation, the supernatant solution was discarded, and the precipitate was redispersed in hexane. The solution was centrifuged at 5000 RPM for 5 minutes to remove aggregated nanocrystals, resulting in the supernatant of long-term colloidally stable solution.

Synthesis of $CsSn_xPb_{1-x}I_3$ perovskite nanocrystals

Cs_2CO_3 (0.26 g, 0.8 mmol), OA (0.8 mL), OLA (0.8 mL) and ODE (24 mL) were loaded into a three-neck flask and degassed under vacuum (~ 1.3 × 10^{-2} mbar) for 2 hours at 110 °C. Afterwards the mixture was heated to 150 °C under N_2 until Cs_2CO_3 fully dissolved. Then the Cs-precursor was heated to the reaction temperature (typically 170 °C) before the injection of SnI_2-PbI_2-TOP precursor (5 mL) which was prepared by heating the mixture of SnI_2 (4 mmol) and PbI_2 (2 mmol, 1.6 mmol, 1 mmol for $CsSn_{0.2}Pb_{0.8}I_3$ NCs, $CsSn_{0.4}Pb_{0.6}I_3$ NCs, and $CsSn_{0.6}Pb_{0.4}I_3$ NCs, respectively) in TOP (5 mL) at 90 °C for 5 hours. The reaction vessel was kept at the injection temperature for 30 s before immersion in an ice-cold water bath. The purification was performed in an argon-filled glovebox. Nanocrystals were purified by an addition of an equal volume of methyl acetate followed by centrifugation at 8000 RPM for 5 minutes. After centrifugation, the supernatant solution was discarded and the precipitate was redispersed in hexane. The solution was centrifuged at 5000 RPM for 5 minutes to remove aggregated nanocrystals, resulting in the supernatant of long-term colloidally stable solution.

Synthesis of Na-doped $CsSn_xPb_{1-x}I_3$ perovskite nanocrystals

Cs_2CO_3 (0.09 g, 0.8 mmol), OA (0.5 mL), OLA (0.5 mL) and ODE (15 mL) were loaded into a three-neck flask and degassed under vacuum (~ 1.3 × 10^{-2} mbar) for 2 hours at 110 °C. Afterwards the mixture was heated to 120 °C under N_2 until Cs_2CO_3 fully dissolved. For sodium doping, after the complete dissolution of Cs_2CO_3, the solution was cooled down to room temperature where sodium acetate trihydrate (0.166 g, 99%, Aldrich) was loaded. The mixture was then heated to 120 °C under nitrogen for 20 min until the sodium salt was completely dissolved. Then the Cs-precursor was heated to the reaction temperature (typically 170 °C) before the injection of SnI_2-PbI_2-TOP precursor (3 mL) which was prepared by heating the mixture of SnI_2 (0.9 g) and PbI_2 (0.3 g) in TOP (3 mL) at 90 °C for 2 hours. The

reaction vessel was kept at the injection temperature for 10 s before immersion in an ice-cold water bath. The purification was performed in an argon-filled glovebox. Nanocrystals were purified by an addition of methyl acetate followed by centrifugation at 8000 RPM for 5 minutes. After centrifugation, the supernatant solution was discarded and the precipitate was redispersed in hexane. The solution was centrifuged at 5000 RPM for 5 minutes to remove aggregated nanocrystals, resulting in the supernatant of long-term colloidally stable solution.

Transmission electron microscopy (TEM) measurements

TEM samples were prepared by putting a small drop of nanoparticle solution onto the carbon coated copper grid in a glovebox. TEM images were recorded using FEI Tecnai F20.

Ultraviolet–visible (UV–Vis) spectroscopy

Steady-state absorption was acquired using an Agilent / Hewlett Packard (HP) 8453 UV-Vis Spectrophotometer with Data System.

Photoluminescence (PL) and photoluminescence quantum efficiency (PLQE) measurements

The steady-state PL and absolute PLQE of $FASnI_3$ nanocrystal samples were measured using an integrating sphere method. A continuous-wave 405-nm diode laser with an excitation power of ~1 mW and a focused beam spot of ~0.3 mm^2 was used to excite the samples. The emission was measured using an Andor iDus *DU420A-BVF* detector.

Transient absorption (TA) spectroscopy measurements (Ti:sapphire laser system)

For transient absorption spectroscopy measurements, the output of a Ti:sapphire amplifier system (Spectra Physics Solstice Ace) operating at 1 kHz and generating ~100-fs pulses was split into the pump and probe beam paths. The 400-nm pump pulses were created by sending the 800-nm fundamental beam of the Solstice Ace through a second harmonic generating (SHG) beta barium borate (BBO) crystal of 1-mm thickness (Eksma Optics). Wavelength tunable pump pulses (e.g., 690-nm pump) were generated in a home-built noncollinear optical parametric amplifier (NOPA). The pump was blocked by a chopper wheel rotating at 500 Hz while a computer operated a mechanical delay stage (Thorlabs DDS300-E/M) to adjust the delay between the pump and the probe. The visible broadband beam (520 – 780 nm) was generated in a home-built noncollinear optical parametric amplifier (NOPA), and the white light was split into two identical beams (probe and reference) by a 50/50 beamsplitter. The reference beam passing through the sample did not interact with the pump, which allows for correcting for any shot-to-shot fluctuations in the probe that would otherwise greatly increase the structured noise in the experiments. Based on this arrangement, small signals with $\Delta T/T$~10^{-5} could be measured. The transmitted probe and

reference pulses were collected with a dual-line array detector driven and read out by a custom-built board (Stresing Entwicklungsbüro).

Transient absorption (TA) spectroscopy measurements (Yb:KGW laser system)

For TA measurements with 500 – 950 nm continuous probe region, a Yb amplifier (PHAROS, Light Conversion) operating at 38 kHz and generating ~200-fs pulses centered at 1030 nm with an output of 14.5 W was used. The ~200 fs pump pulse was provided by a TOPAS OPA. The white light supercontinuum probe was generated by sending in a small portion of the 1030-nm fundamental to a YAG crystal (4 mm). The transmitted probe was imaged using a Si photodiode array (Stresing S11490).

Time-correlated single photon counting (TCSPC) measurements

The nanocrystal solutions in 1-mm cuvettes were photo-excited using a 407-nm pulsed laser with a pulse width <200 ps, at a repetition rate of 40 MHz or 10 MHz. Photons emitted from the sample were collected by a Si-based single-photon avalanche photodiode. The instrument response function has a lifetime of ~0.2 ns. A 420-nm long-pass filter was used to screen out any scattered laser signal in the optical path.

Notes and Figures

Note S1. Calculation of pump fluence

Fluence per pulse can be calculated by Fluence (per pulse) = $P/(f \times S)$, where P is the power of the pump which can be measured by a power meter, f is the repetition rate which is f = 500 Hz for our TA with Ti:sapphire laser system and f = 19 kHz for our TA with Yb:KGW laser system, $S = \pi r^2$ is the area of the pump beam spot (2r: effective beam diameter measured by the Thorlabs beam profiler).

Carrier density is proportional to the photons absorbed by the material, which can be calculated by:
The number of photons absorbed (per pulse) = Fluence (per pulse)×$(1-10^{-\mathrm{OD}})/h\nu$, where OD is the optical density of the material measured by UV-Vis spectroscopy, h is the Planck constant, ν is the frequency of light.

Note S2. Determination of absorption cross-section of nanocrystals

Based on a Poisson distribution of early time nanocrystal occupancies, the probability of a nanocrystal containing i excitons is given by $p_i = (\langle N\rangle^i/i!)\exp(-\langle N\rangle)$, where $\langle N\rangle = \sigma j$ is the average number of excitons per nanocrystal (σ: the absorption cross-section of the nanocrystals, j: pump fluence). After the completion of multiexciton recombination (~200 ps), each photoexcited nanocrystal contains only one electron-hole pair independent of its initial occupancy. So the amplitude of the TA signal is directly proportional to the total number of the photoexcited nanocrystals, which could be presented as $I_{\mathrm{TA}} \propto (1-p_0) = (1-\mathrm{e}^{-\langle N\rangle}) = (1-\mathrm{e}^{-\sigma j})$. The absorption cross-section was determined from the fitting parameters. In our work, the absorption cross-sections are 1.34×10^{-14} cm^2 ($MASnI_3$ NCs), 1.61×10^{-14} cm^2 ($MASn_{0.9}Pb_{0.1}I_3$ NCs), 1.31×10^{-14} cm^2 ($MASn_{0.75}Pb_{0.25}I_3$ NCs), 1.40×10^{-14} cm^2 ($MASn_{0.6}Pb_{0.4}I_3$ NCs), 1.92×10^{-14} cm^2 ($MASn_{0.3}Pb_{0.7}I_3$ NCs),and 1.81×10^{-14} cm^2 ($MAPbI_3$ NCs) at 400 nm.

Note S3. Calculation of the initial carrier density

The absolute carrier density can be determined by $n_0 = \langle N\rangle/V_{\mathrm{NC}}$ where $\langle N\rangle$ is the average number of excitons per nanocrystal and V_{NC} is the nanocrystal volume.

Note S4. Discussion on models used to determine charge-carrier temperature

In our work, the carrier temperature is determined by fitting the high-energy tail of the TA spectra with a Boltzmann distribution model, $f \sim \exp(E–E_f)/k_BT$ (E_f, fermi energy, k_B, the Boltzmann constant). This method has been widely used in perovskites[1-8] and other semicondoctors such as GaAs[9-12] and CdS[13], where the extracted temperature was used to study carrier cooling. However, the assumption that hot carriers obey Bolzman distribution is based on a constant density of state with energy. In real cases, carrier distribution is also shaped by the variation of density of states with energy[14,15], where parabolicity has been confirmed for both lead-halide and tin-halide perovskites[16,17], with the joint density of states (JDOS) for direct band-to-band transitions between parabolic bands given as JDOS ~ $(E–E_{gap})^{1/2}$. Previous reports showed that JDOS accounts for the main inconsistency between the actual carrier temperature and the fitted value from a Boltzmann distribution model. Ignoring the square-root density of states given by the parabolic band structure has been confirmed to give a small overestimation of carrier temperature at high temperatures (>400 K), where the residual is non-divergent as carrier temperature increases. As a result, the variation in the JDOS is not important compared with the change in the Boltzmann distribution.

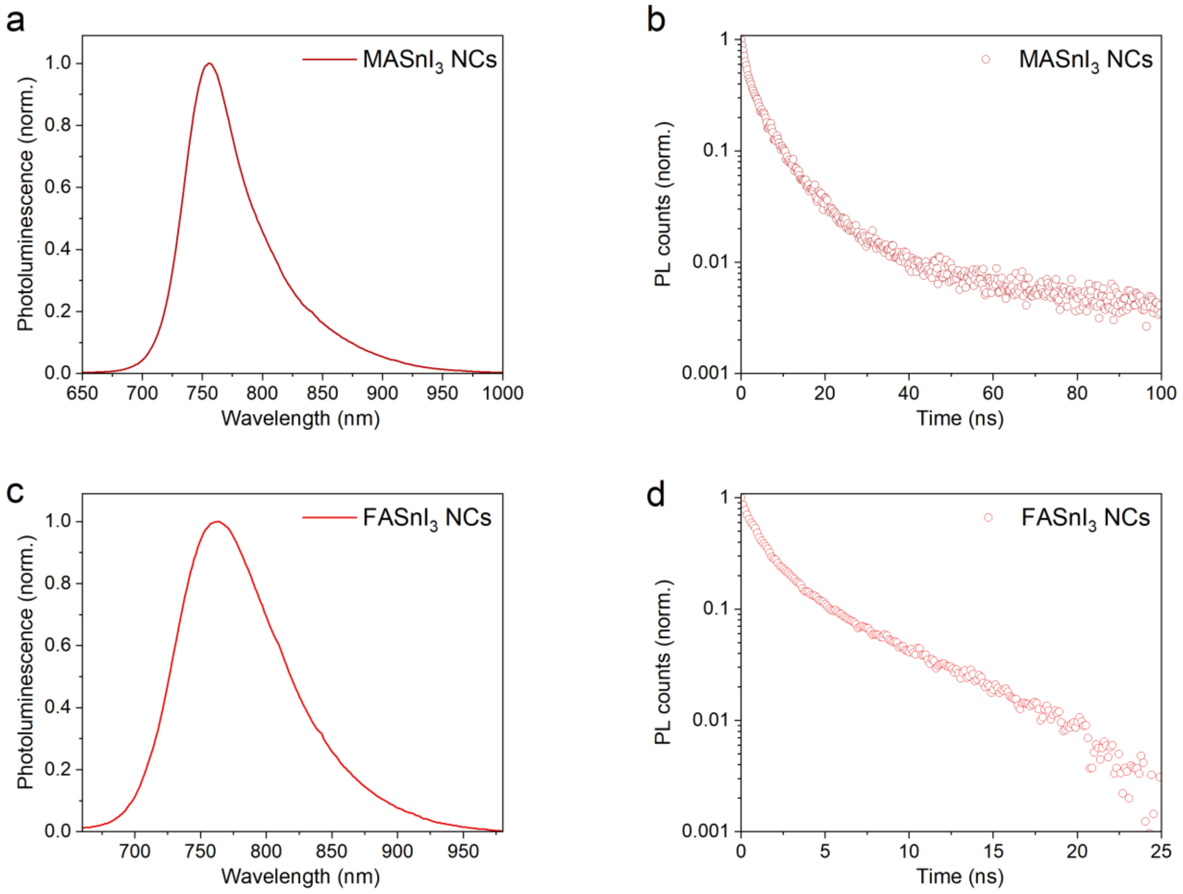

Figure S1. Steady-state photoluminescence and time-resolved photoluminescence of (a, b) $MASnI_3$ NCs (~3 nm) and (c, d) $FASnI_3$ NCs (~12 nm).

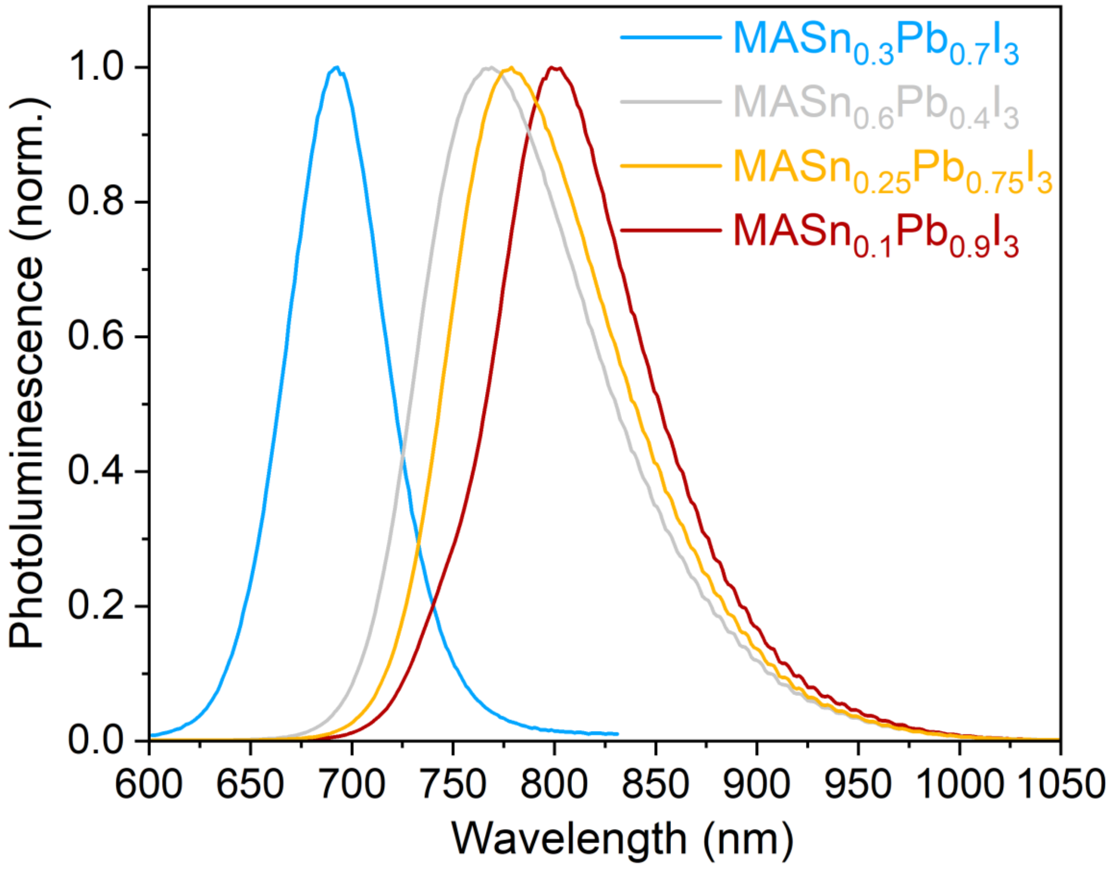

Figure S2. Steady-state photoluminescence of $MASn_xPb_{1-x}I_3$ NCs (~3 nm) under a 405-nm pump.

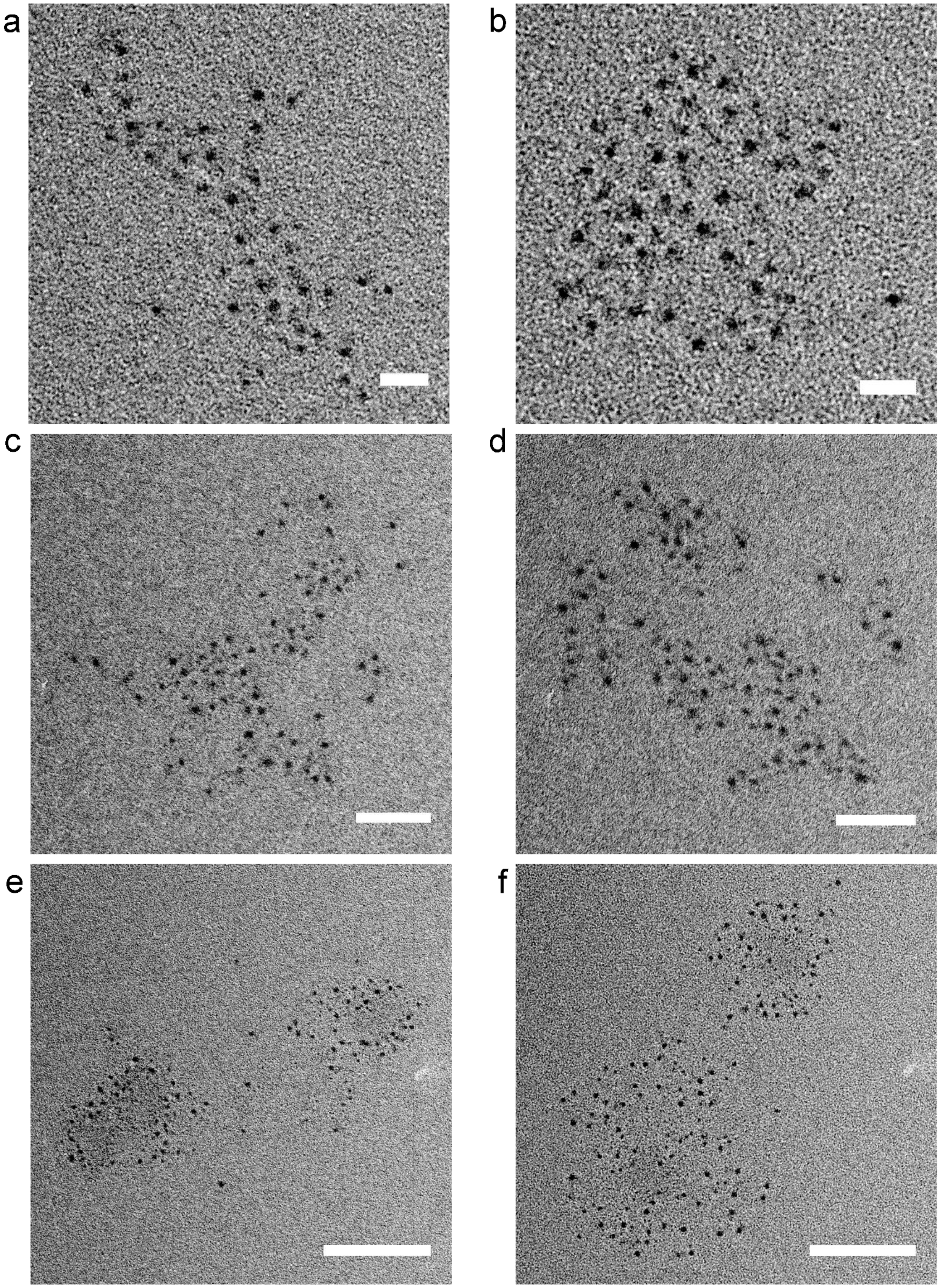

Figure S3. TEM images of $MAPbI_3$ nanocrystals synthesized at room temperature using a LARP method (scale bars, (a, b) 20 nm, (c, d) 50 nm, (e, f) 100 nm).

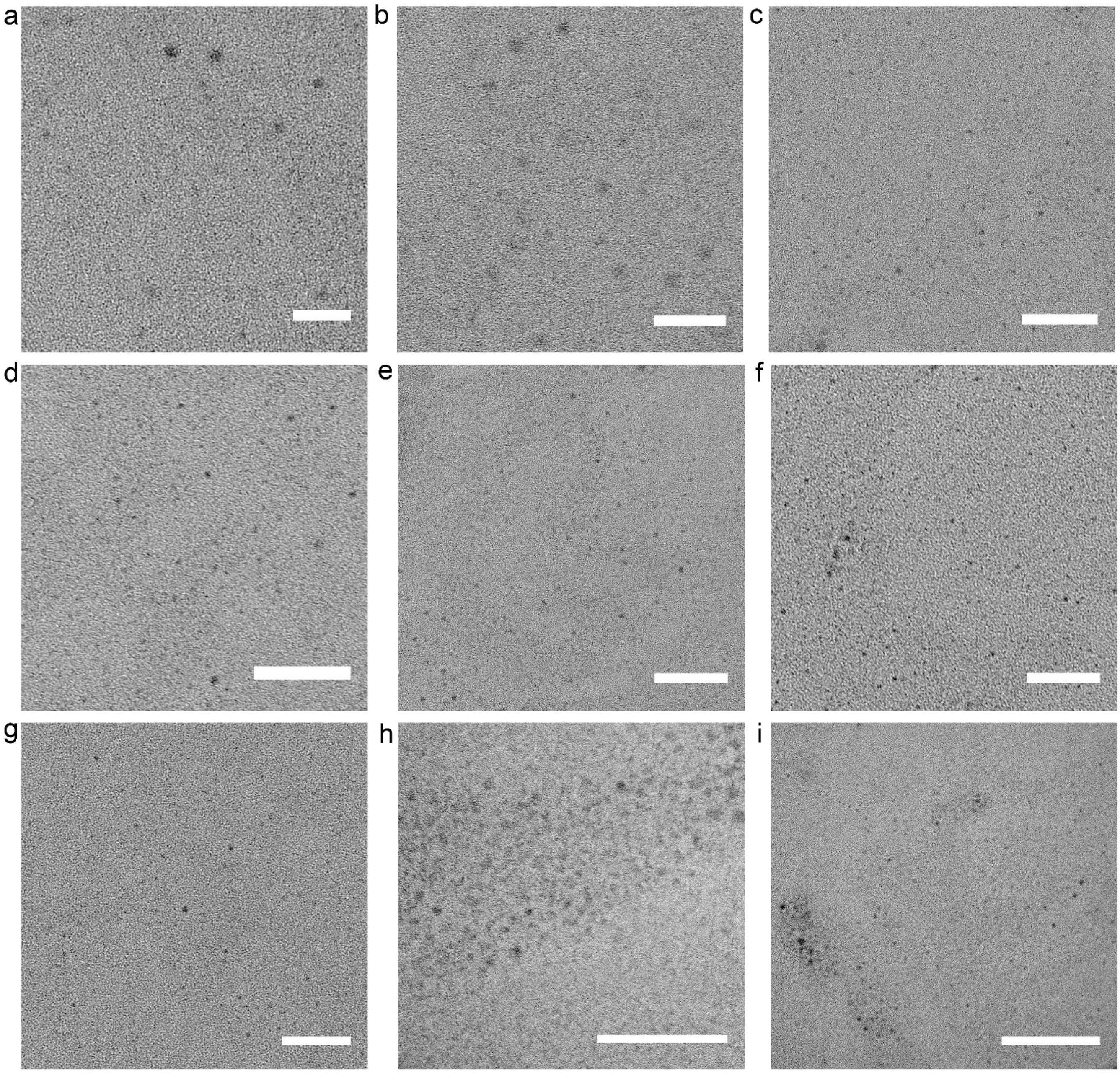

Figure S4. TEM images of $MASn_xPb_{1-x}I_3$ nanocrystals synthesized at room temperature using a LARP method (scale bars, (a, b) 20 nm, (c-h) 50 nm, (i) 100 nm).

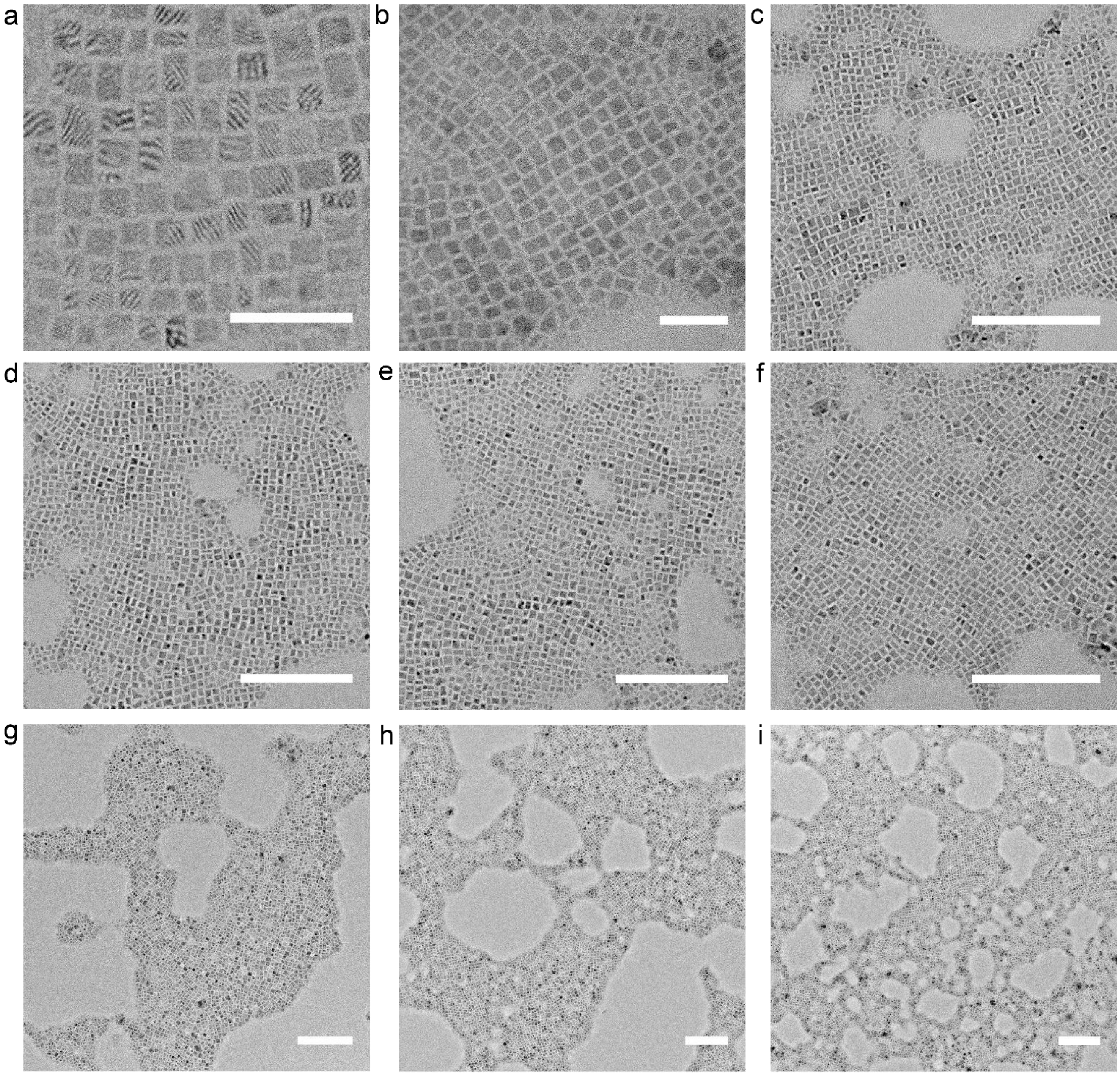

Figure S5. TEM images of $MAPbI_3$ nanocrystals synthesized at 60 °C with a reaction time of 30 s using a LARP method (scale bars, (a, b) 50 nm, (c-i) 200 nm).

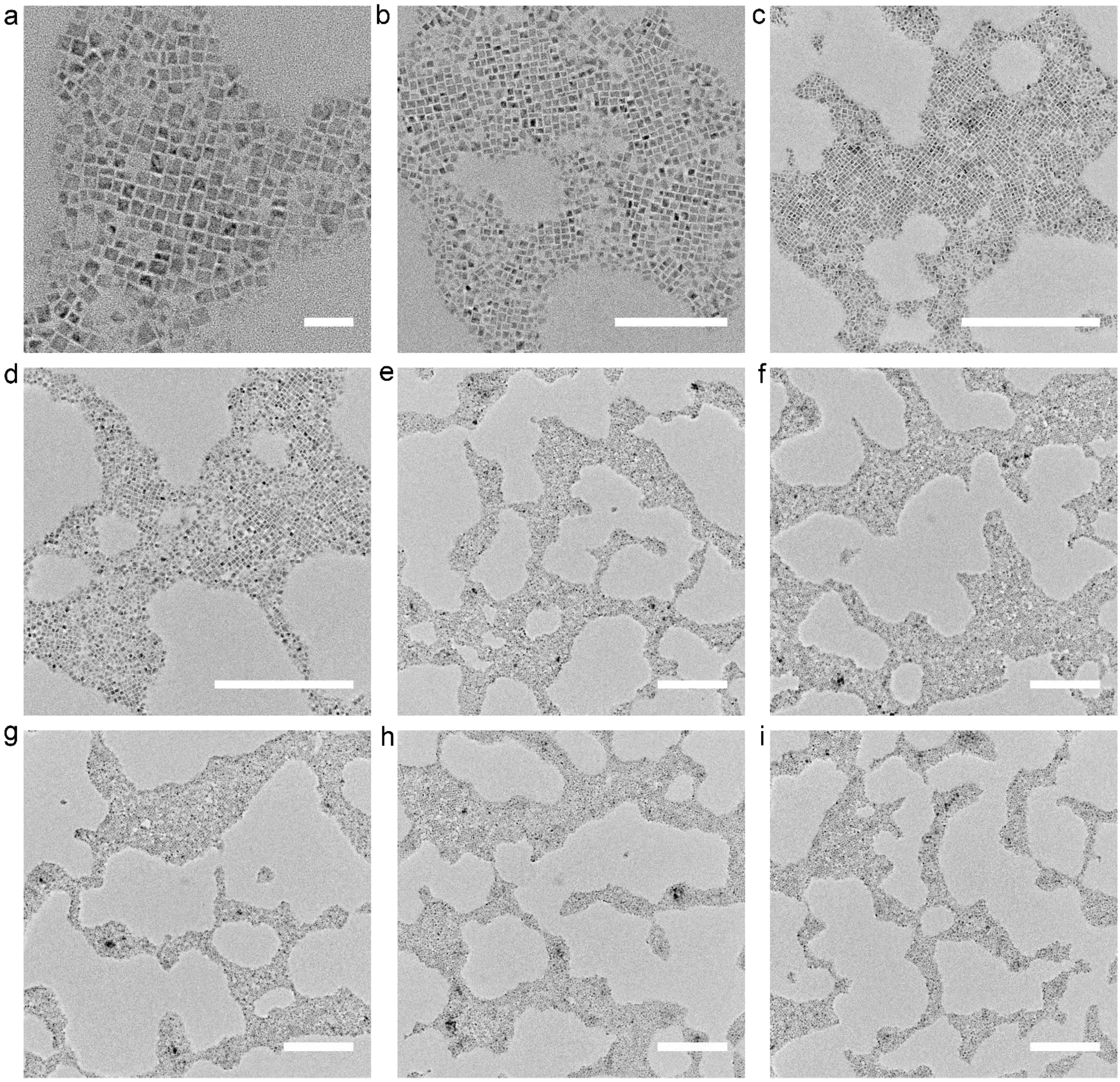

Figure S6. TEM images of $MASn_xPb_{1-x}I_3$ nanocrystals synthesized at 60 °C with a reaction time of 30 s using a LARP method (scale bars, (a) 50 nm, (b) 200 nm, (c-i) 500 nm).

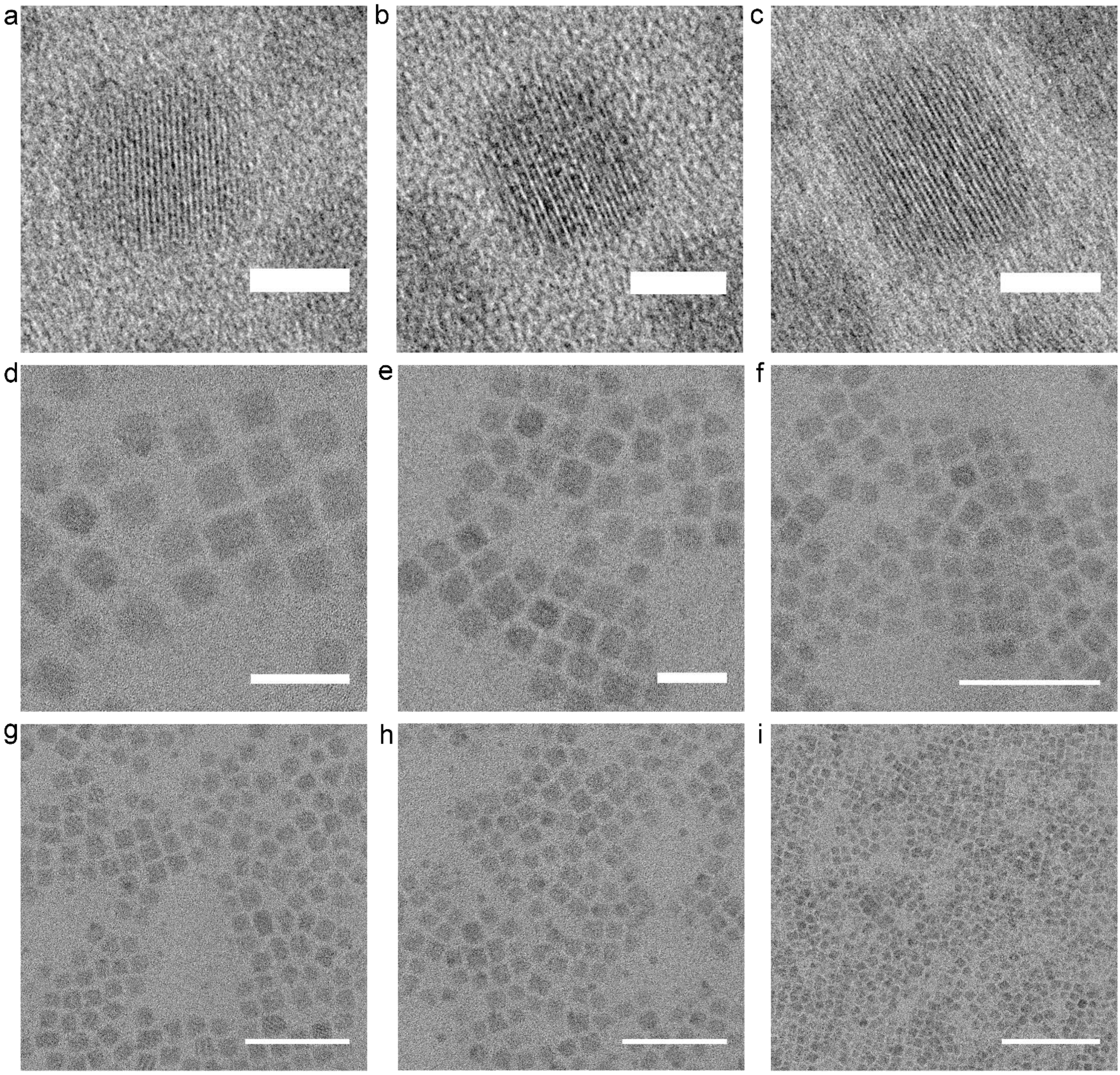

Figure S7. TEM images of $CsSn_xPb_{1-x}I_3$ nanocrystals synthesized at 170 °C (scale bars, (a-c) 5 nm, (d, e) 20 nm, (f-h) 50 nm, (i) 100 nm).

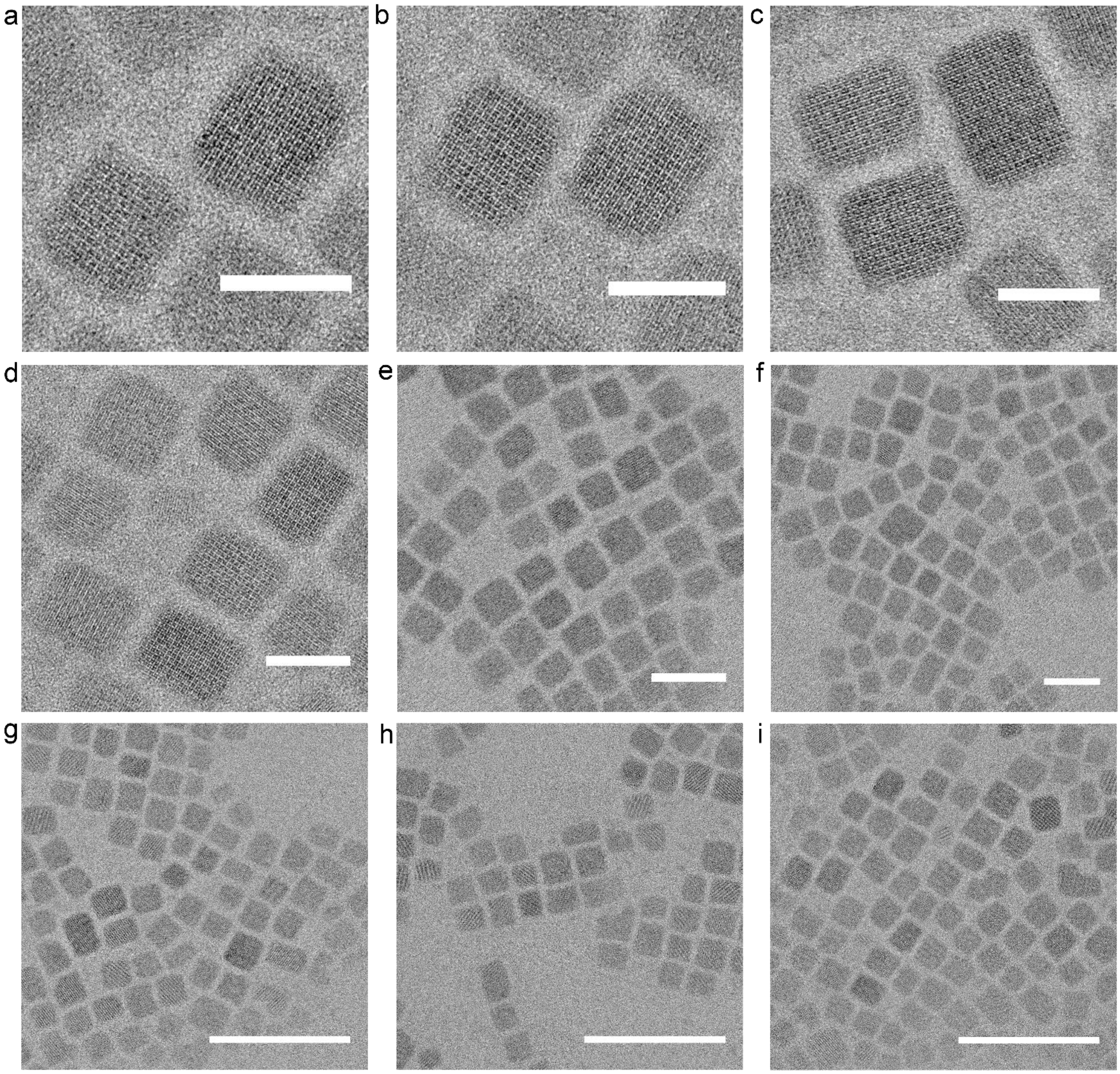

Figure S8. TEM images of Na-doped $CsSn_xPb_{1-x}I_3$ nanocrystals synthesized at 170 °C (scale bars, (a-d) 10 nm, (e, f) 20 nm, (g-i) 50 nm).

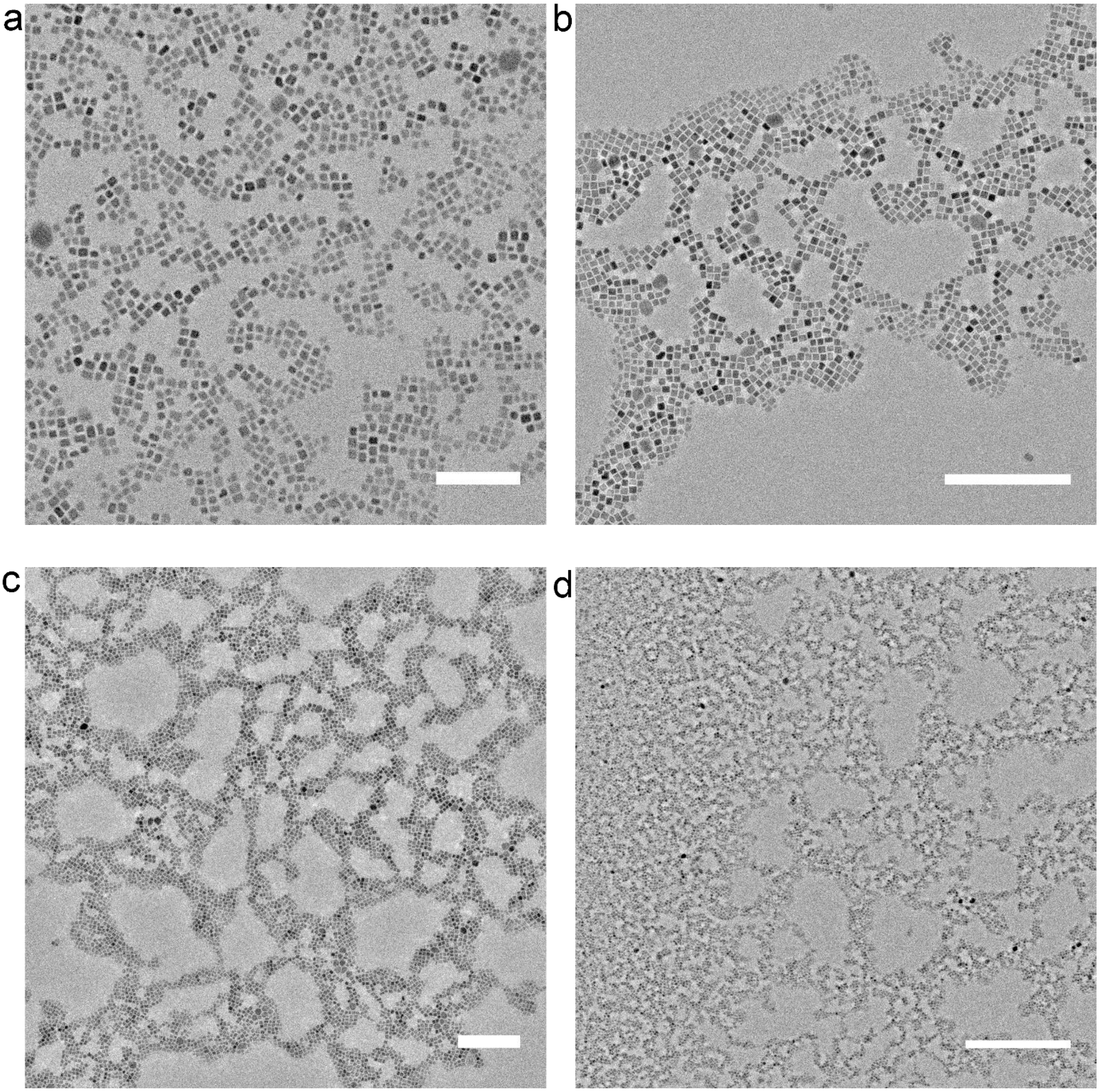

Figure S9. TEM images of Na-doped $CsSn_xPb_{1-x}I_3$ nanocrystals synthesized at 170 °C with a large filed of view (scale bars, (a) 100 nm, (b, c) 200 nm, (d) 500 nm).

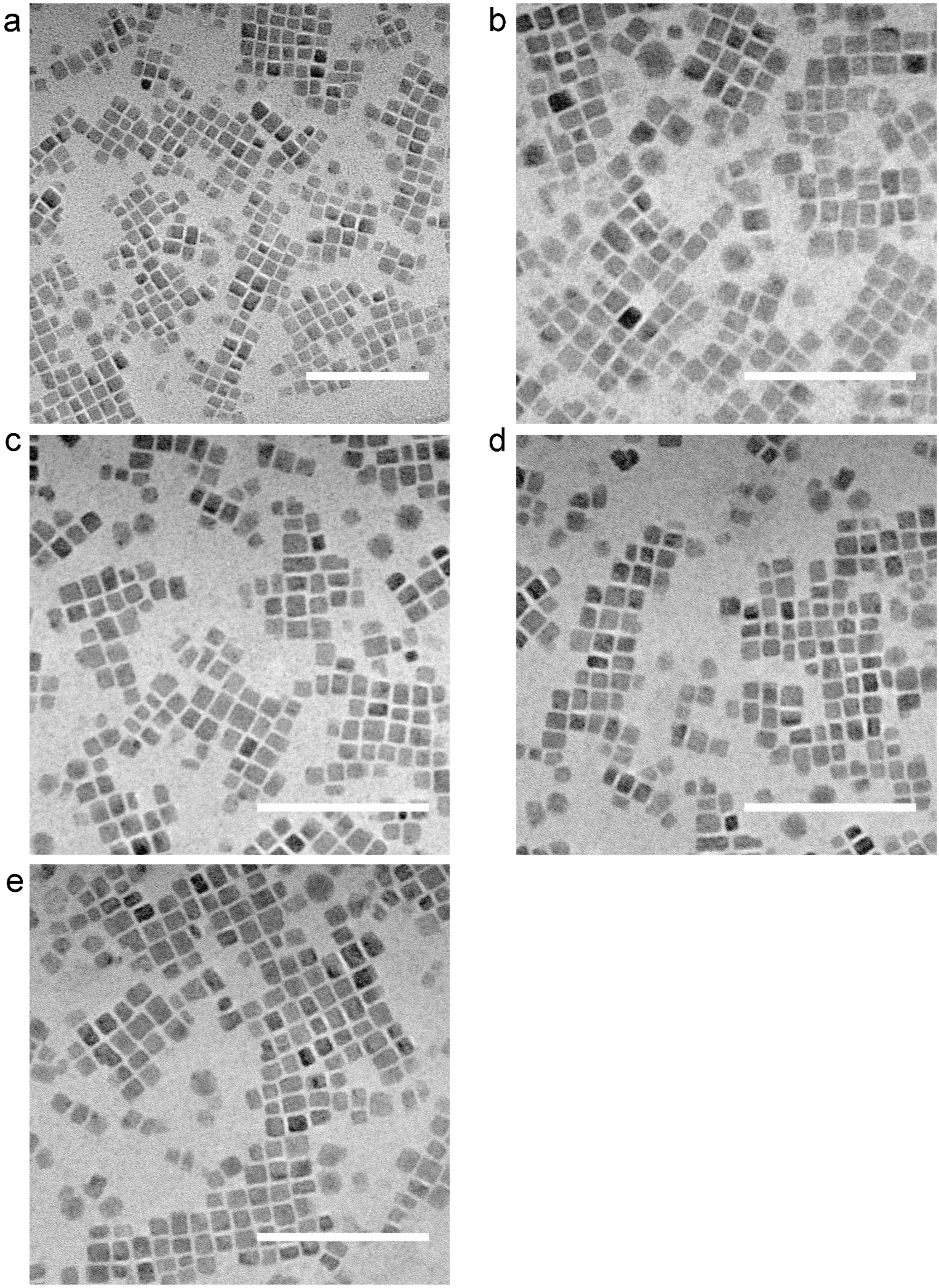

Figure S10. TEM images of $CsSn_{0.4}Pb_{0.6}Br_3$ nanocrystals synthesized at 170 °C (scale bars, 100 nm).

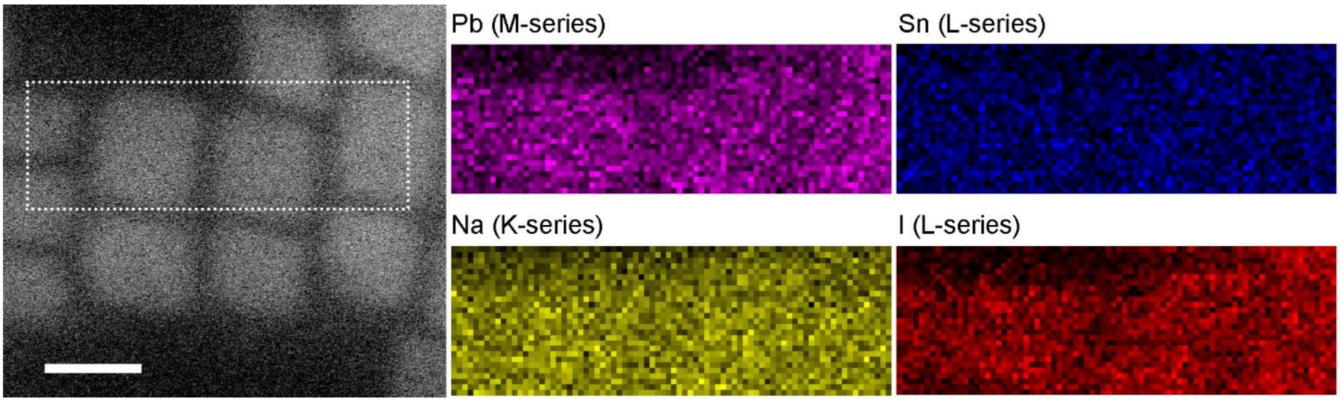

Figure S11. STEM image and EDS mapping of Na-doped $CsSn_xPb_{1-x}I_3$ nanocrystals synthesized at 170 °C (scale bar, 10 nm).

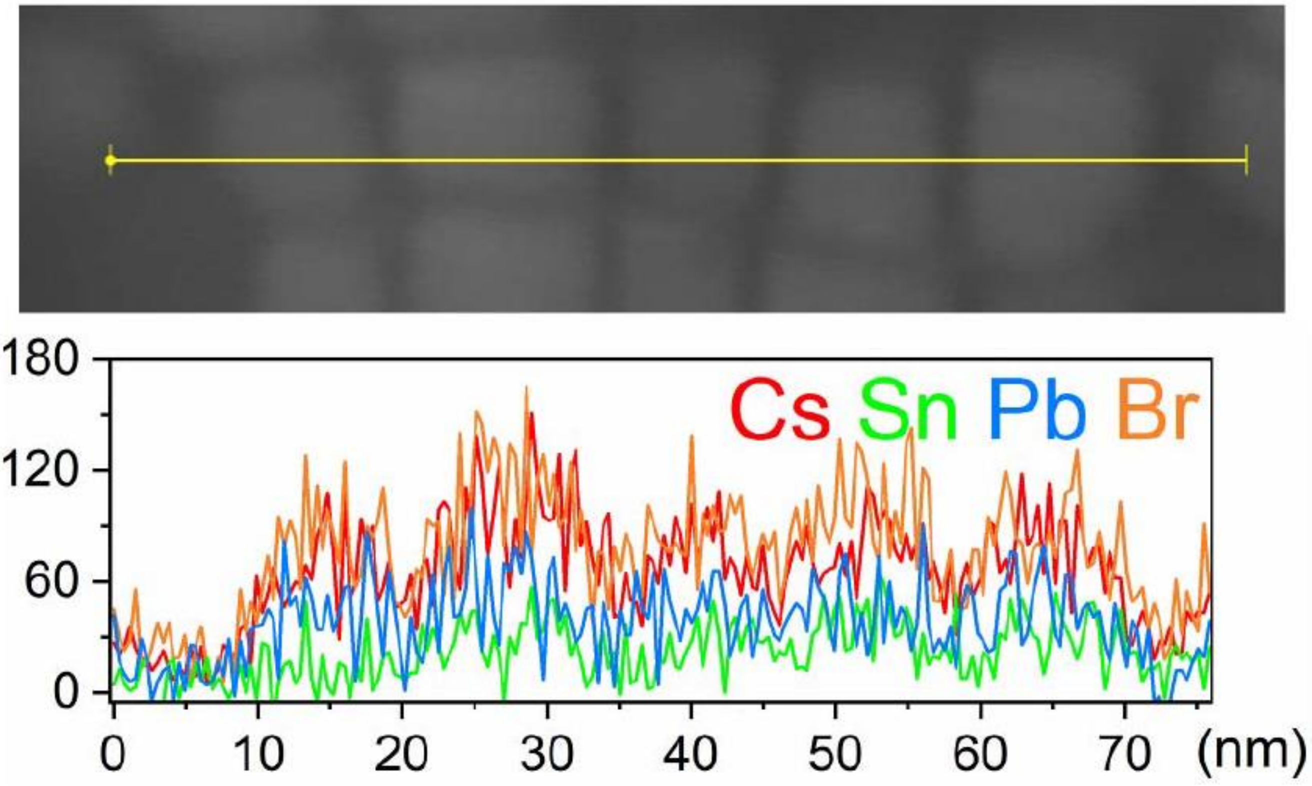

Figure S12. EDS line scan of $CsSn_{0.4}Pb_{0.6}Br_3$ NCs synthesized at 170 °C. According to the EDS line scan, the Cs, Pb, Sn and Br elements share the same trend in the change of counts per seconds. Every peak of these lines corresponds to a middle position of a nanocrystal while each minimum of these lines indicates the gap between the two neighboring nanocrystals. The results confirm that each nanocrystal is an alloy of Sn and Pb, containing similar composition. The atom percentages of Cs, Sn, Pb and Br are ~24%, ~9%, ~13% and ~54% respectively, corresponding to a ratio of Cs:Sn:Pb:Br ~ 6 : 2 : 3 : 13, broadly consistent with the perovskite chemical formula of $CsSn_{0.4}Pb_{0.6}Br_3$.

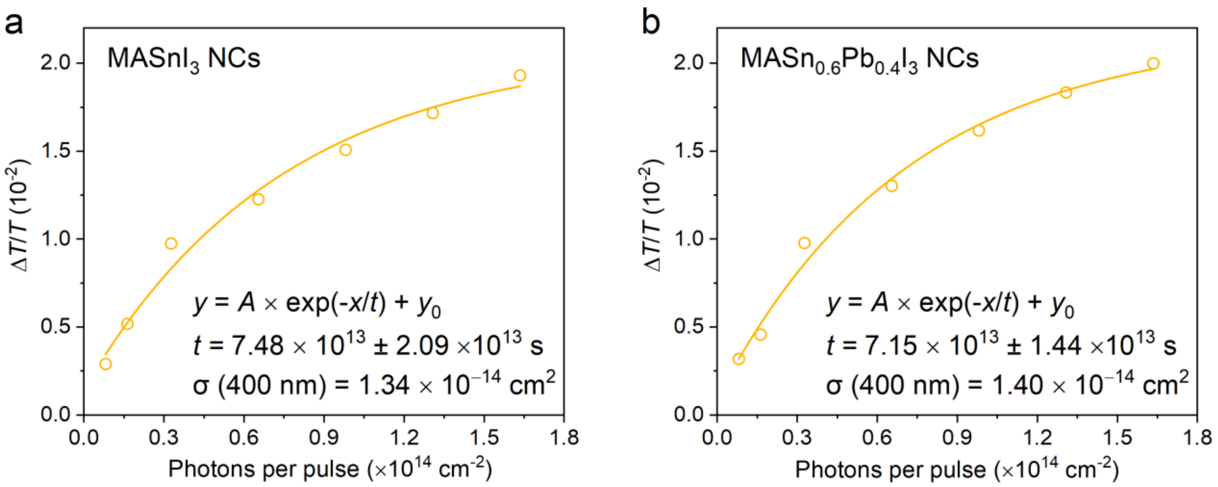

Figure S13. Determination of the absorption cross-section of $MASnI_3$ NCs and $MASn_xPb_{1-x}I_3$ NCs.

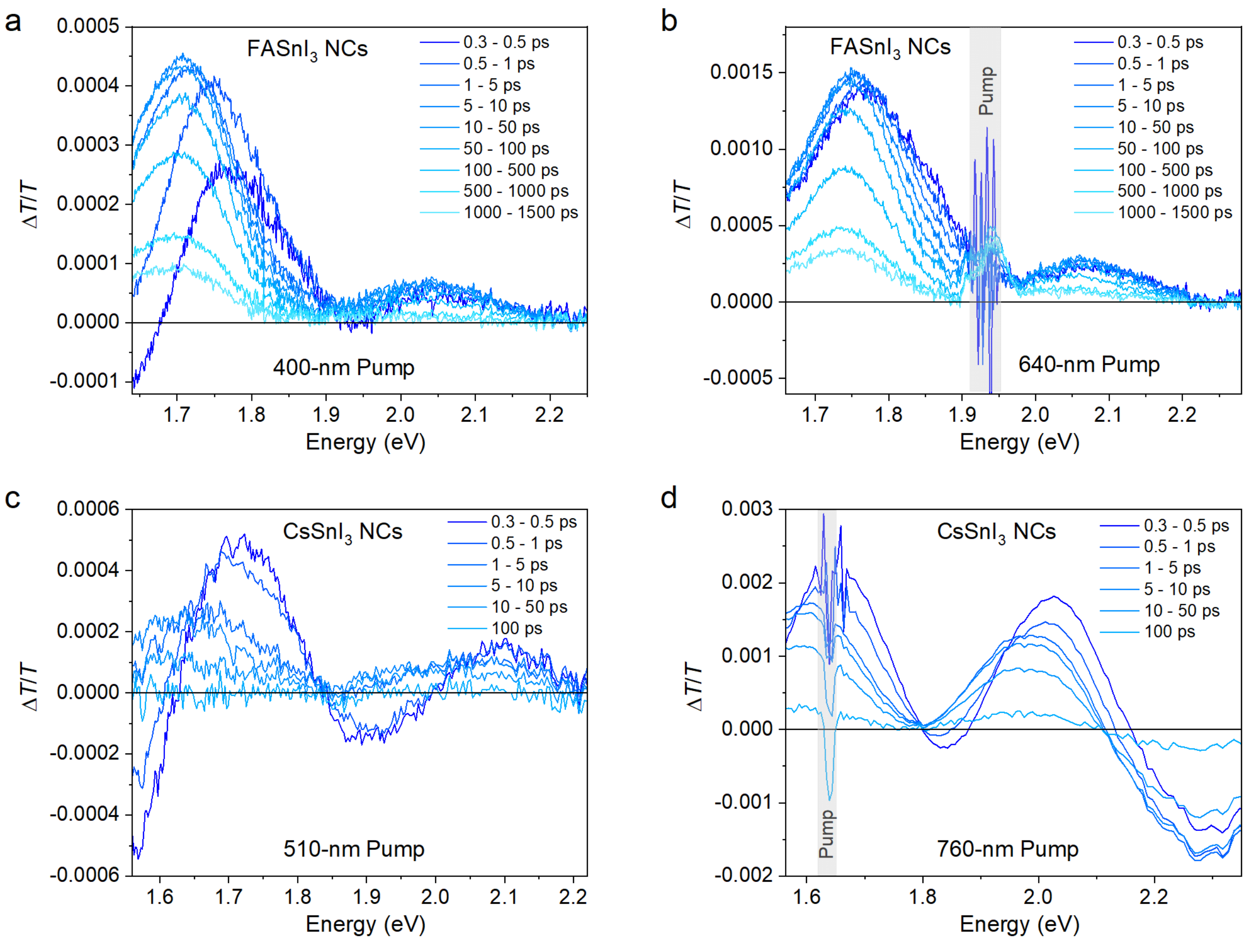

Figure S14. TA spectra of $FASnI_3$ NCs under (a) a 400-nm pump and (b) a 640-nm pump. TA spectra of $CsSnI_3$ NCs under (c) a 510-nm pump and (d) a 760-nm pump.

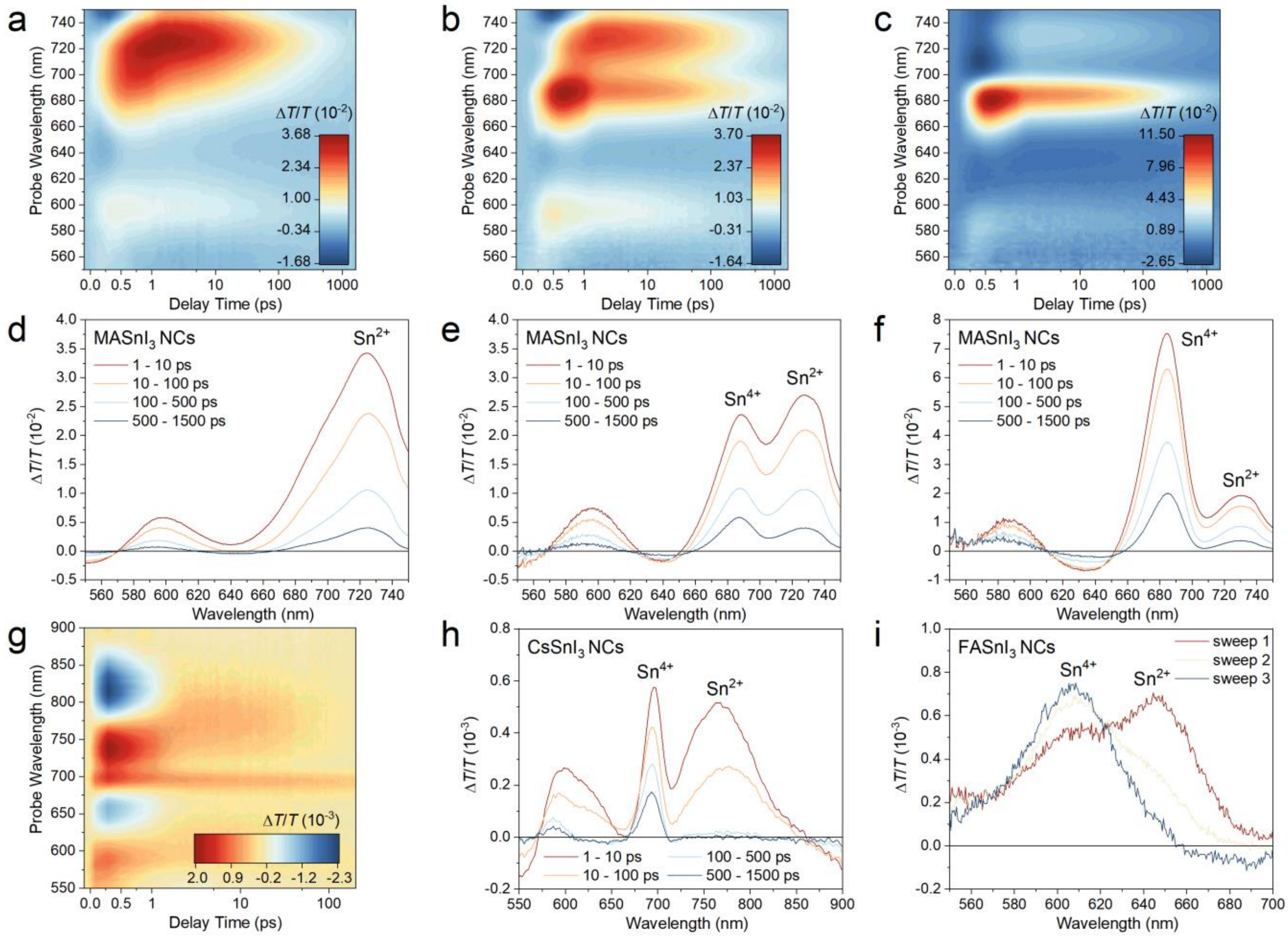

Figure S15. Degradation processes of $MASnI_3$ NCs, $FASnI_3$ NCs and $CsSnI_3$ NCs. (a-f) TA maps and TA spectra of $MASnI_3$ NCs during the degradation process under a 400-nm pump (a&d, fresh $MASnI_3$ NC, b&e, c&f, degraded $MASnI_3$ NCs). (g, h) TA map and TA spectra of degraded $CsSnI_3$ NCs under a 515-nm pump. (i) Early time (0.5 to 2 ps average) TA spectra of $FASnI_3$ NCs under a 400-nm pump.

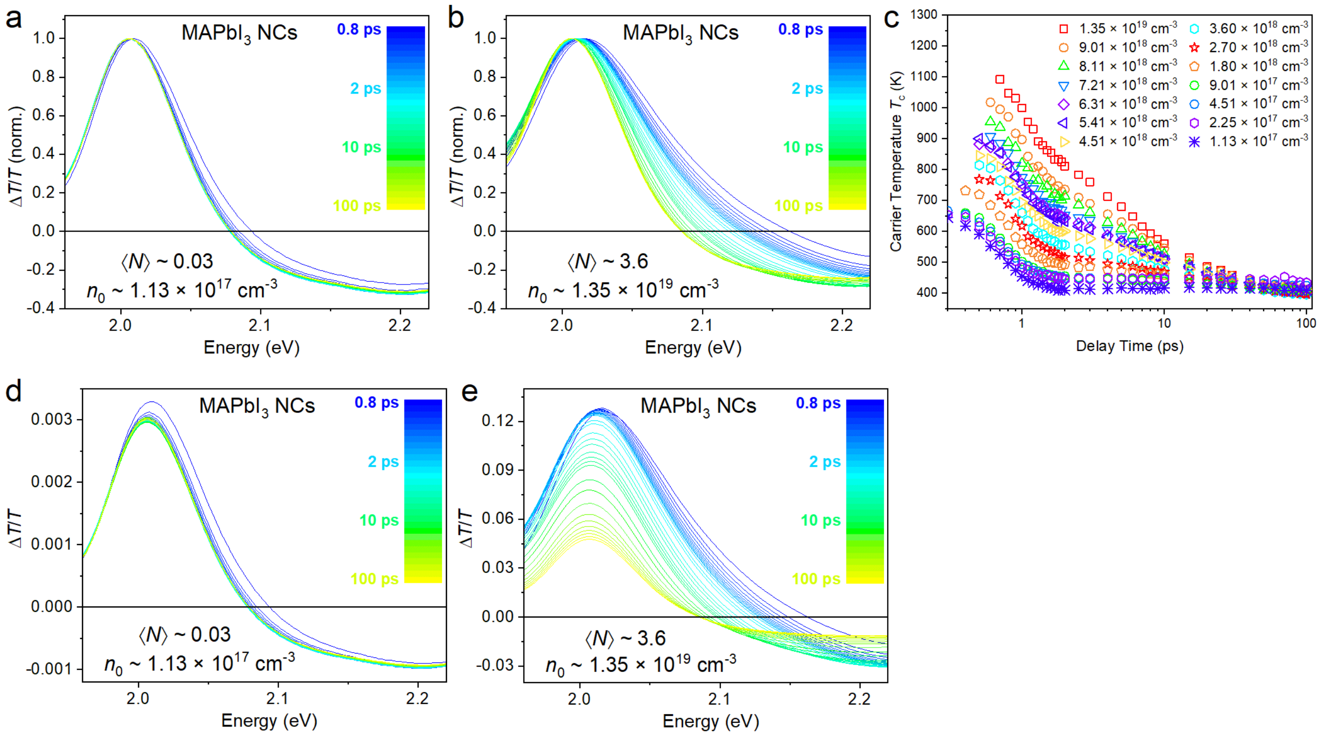

Figure S16. Transient absorption spectroscopy of $MAPbI_3$ NCs (~3 nm) under different pump fluences. (a, d) Normalized and non-normalized TA spectra (0.8 ps to 100 ps) of $MAPbI_3$ NCs under a low fluence pump. (b, e) Normalized and non-normalized TA spectra (0.8 ps to 100 ps) of $MAPbI_3$ NCs under a high fluence pump. (c) Hot carrier temperature extracted from TA spectra for different initial carrier densities.

Figure S16 shows excitation-intensity-dependent TA measurements of $MAPbI_3$ NCs. For a low-fluence pump corresponding to an injected carrier density of 1.13×10^{17} cm^{-3} (Figure S16a), no obvious change in the shape of the high-energy tail can be observed after 1 ps, whereas for a high-fluence pump with injected carrier density of 1.35×10^{19} cm^{-3} (Figure S16b), a narrowing of the broad bleach from 0.8 ps to 100 ps is observed, indicating a slow cooling mediated by the hot phonon bottleneck effect. Figure S16c shows the time-dependent carrier temperatures under different injected carrier densities. A slow decay occurs at carrier densities higher than ~10^{18} cm^{-3}, consistent with the previous report for bulk $MAPbI_3$ perovskites where a threshold carrier density was determined to be ~5×10^{17} cm^{-3} for the hot phonon bottleneck in bulk $MAPbI_3$ perovskites[2]. Thus, we see that $MAPbI_3$ NCs exhibit a hot phonon bottleneck effect on the timescale of ~10 ps under high-fluence pump, stronger than the effect for $MASnI_3$ NCs (~7 ps). One reason for the shortened hot carrier lifetime is the presence of high-density deep traps in Sn-based perovskites, evidenced by the significant decline in PLQE from ~34% ($MAPbI_3$ NCs) to ~0.06% ($MASnI_3$ NCs). These defect traps strongly couple with hot carriers and provide additional relaxation paths, leading to increased hot carrier cooling rate in $MASnI_3$ NCs compared to lead counterparts.

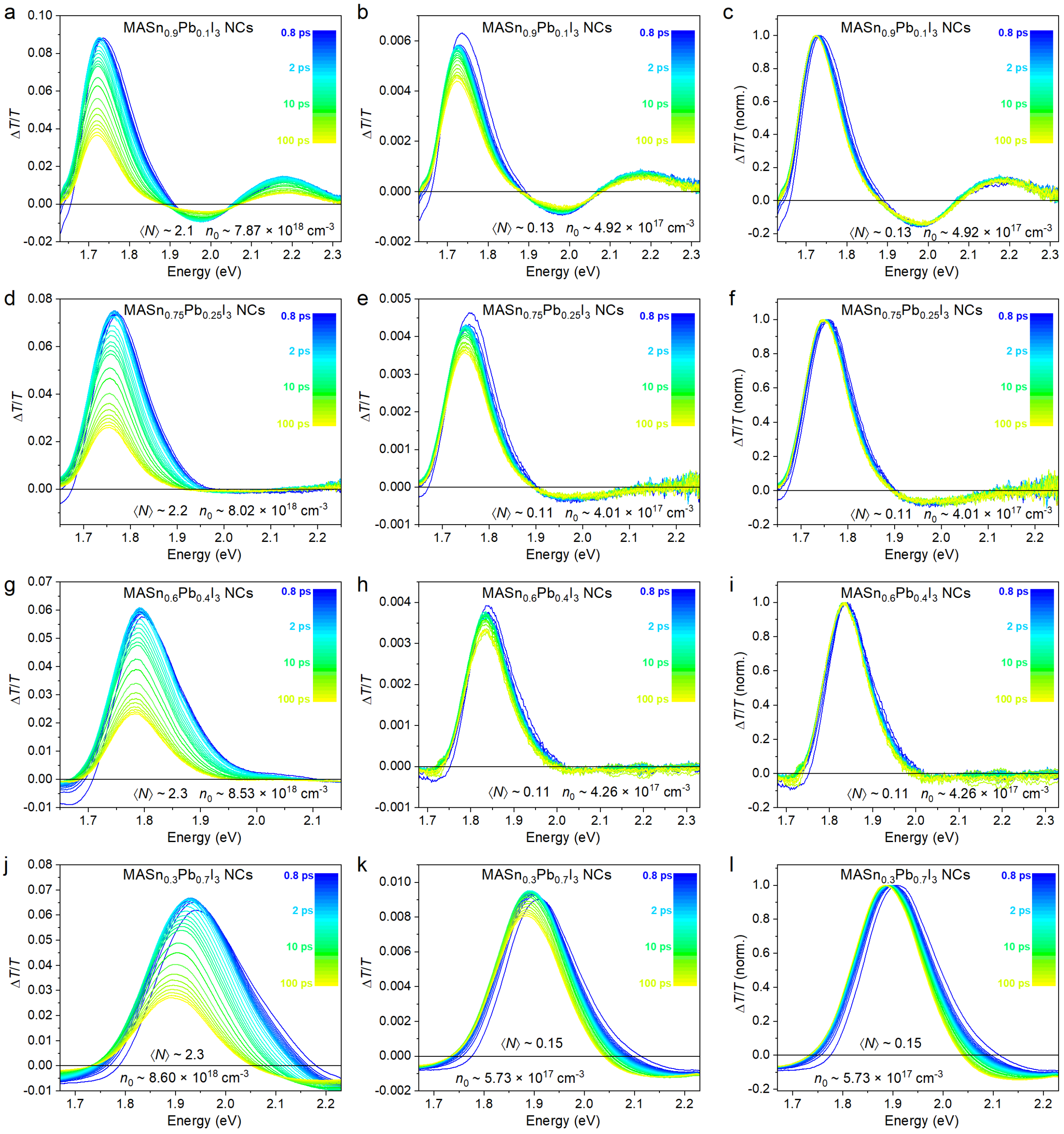

Figure S17. Non-normalized TA spectra under a high-fluence pump, non-normalized and normalized TA spectra under a low-fluence pump for (a-c) $MASn_{0.9}Pb_{0.1}I_3$ NCs, (d-f) $MASn_{0.75}Pb_{0.25}I_3$ NCs, (g-i) $MASn_{0.6}Pb_{0.4}I_3$ NCs, and (j-l) $MASn_{0.3}Pb_{0.7}I_3$ NCs.

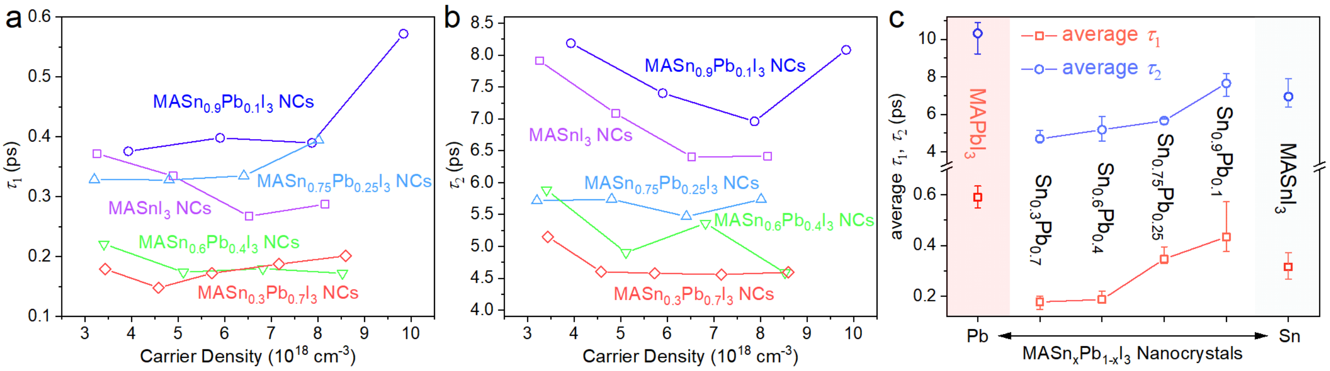

Figure S18. (a, b) Fitting parameters, (a) τ_1 and (b) τ_2, extracted from the bi-exponential decay of time-dependent hot carrier temperature in $MASn_xPb_{1-x}I_3$ NCs. (c) Average τ_1 and τ_2 for $MASn_xPb_{1-x}I_3$ NCs.

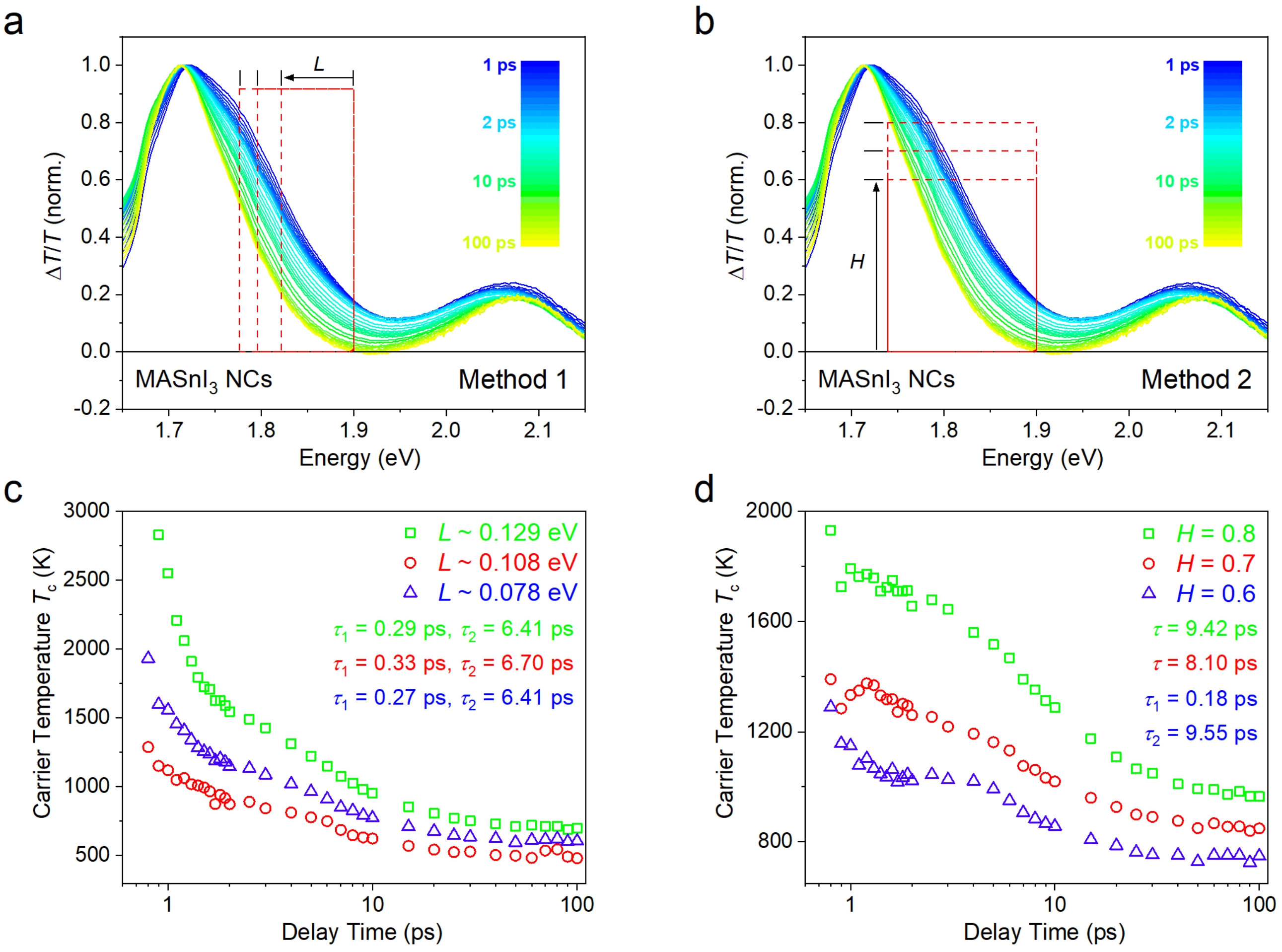

Figure S19. (a, b) TA spectra of $MASnI_3$ nanocrystals with different methods of choosing ROIs for determination of carrier temperature (injected carrier density, 8.16×10^{18} cm^{-3}), with (c, d) corresponding time-dependent carrier temperature T_c with different values of L and H. There are two common methods for choosing the region of interest (ROI) for fitting with Boltzmann distribution when determining the carrier temperature. Method 1 involves choosing an ROI of fixed length, L, along the x-axis (S19a). Method 2 defines an ROI from a fixed height H (spectra normalized in y-axis) to a certain energy on the x-axis near the trough of the spectra to avoid the upward curvature (S19b). Figure S19c shows the fitted T_c according to Method 1 with L ranging from 0.078 eV to 0.129 eV. As L increases, the absolute value of fitted T_c increases gradually. The curves with different L values can all be fitted using bi-exponential decay with very similar τ_1 and τ_2 of ~0.3 ps and ~6.5 ps. This shows that although the length of ROI may affect the absolute value of fitted carrier temperature for NCs (where a wide ROI window is more sensitive than a narrow one), the trend of the curve stays quite similar. Figure S19d shows the fitted T_c according to Method 2 with H ranging from 0.6 to 0.8. Similar to Method 1, the absolute value of fitted T_c increases as H increases. For $H = 0.6$, the curve can be fitted using bi-exponential decay and the fitted τ_1 and τ_2 are of the same order as the Method 1 (sub-picosecond and several picoseconds respectively). However, for $H = 0.7$ and 0.8, the time-dependent carrier temperature curves can no longer be fitted using bi-exponential decay as the information about sub-picosecond cooling is missing. This instability of fitted

carrier temperature with different H value is also observed in Sn-Pb binary perovskite nanocrystals, see Figure S20. As a result, Method 1 is used in the main text as a standard procedure for the determination of τ_1 and τ_2, which makes the results reliable and comparable between different nanocrystals.

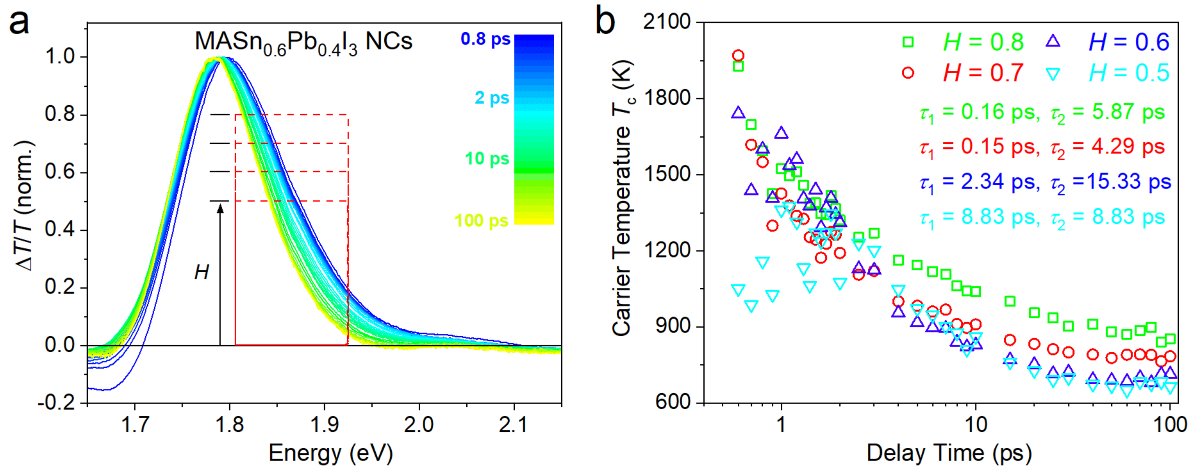

Figure S20. (a) TA spectra of $MASn_{0.6}Pb_{0.4}I_3$ nanocrystals with ROIs according to Method 2. (b) The corresponding time-dependent carrier temperature T_c with different values of H. As H increases from 0.5 to 0.8, the fitted values of τ_1 and τ_2 are greatly affected, indicating that determination of cooling time from Method 2 relies dramatically on the ROI chosen.

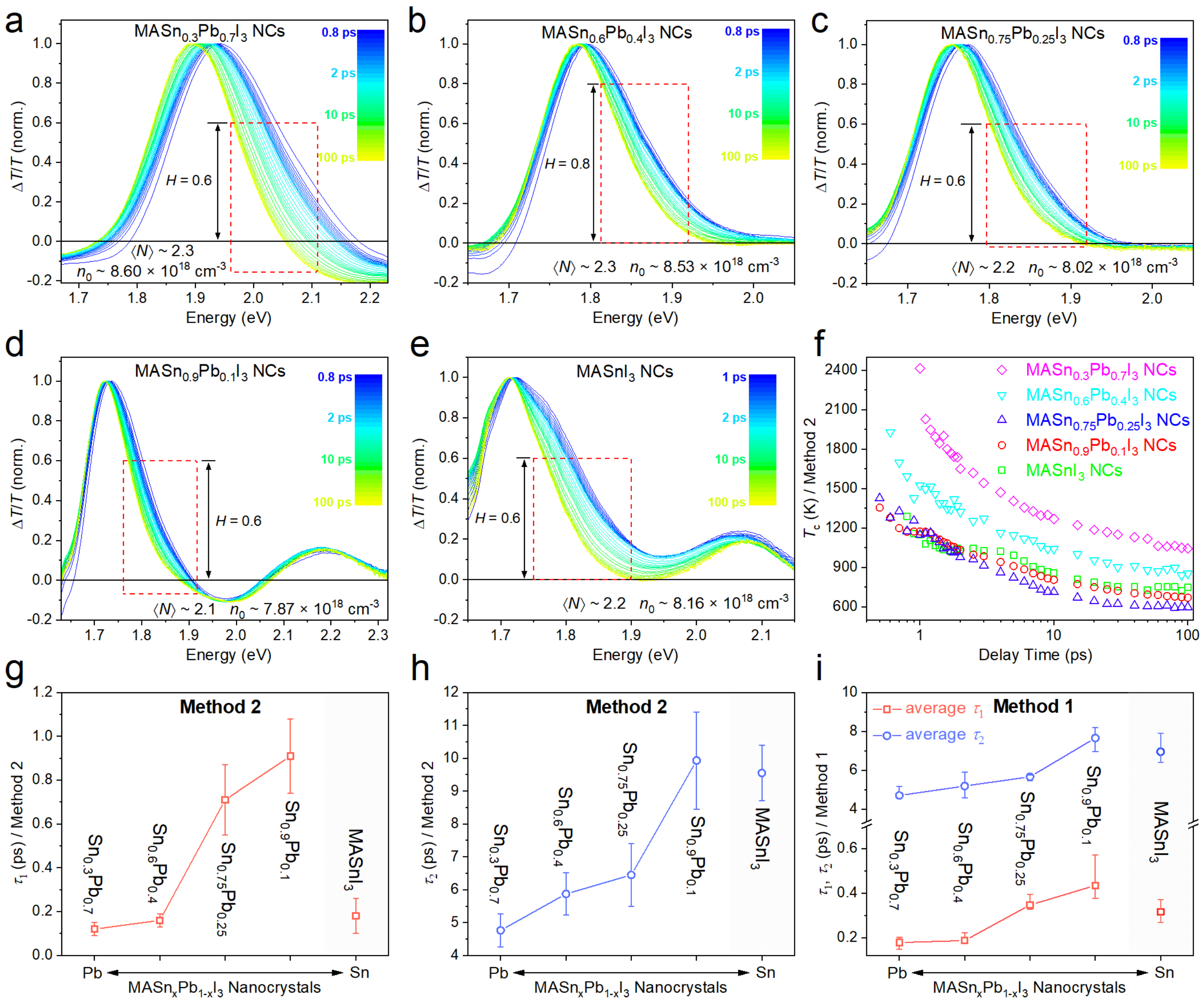

Figure S21. TA spectra under high injected carrier densities (0.8 ps to 100 ps) of (a) $MASn_{0.3}Pb_{0.7}I_3$ NCs, (b) $MASn_{0.6}Pb_{0.4}I_3$ NCs, (c) $MASn_{0.75}Pb_{0.25}I_3$ NCs, (d) $MASn_{0.9}Pb_{0.1}I_3$ NCs and (e) $MASnI_3$ NCs. (f) The corresponding time-dependent carrier temperature curves extracted from the ROIs according to Method 2 (red squares in (a–e)). (g, h) Fitting parameters, τ_1 and τ_2, from the bi-exponential decay for curves extracted from Method 2 (error bars belong to the fitting model.). (i) Average fitting parameters, τ_1 and τ_2, from the bi-exponential decay for curves extracted from Method 1 (error bars take the maximum and minimum values into consideration). The comparison of hot carrier cooling (using Method 1) between nanocrystals with different Sn-Pb ratio is shown in the main text. Figure S21 shows that the cooling trend observed via Method 1 is further confirmed using Method 2. We set H = 0.6 as a standard for the Method 2 approach (H = 0.8 for $MASn_{0.6}Pb_{0.4}I_3$ NCs, see Figure S20), and the spectra with ROIs of different nanocrystals are shown in Figure S21a–e. Figure S21f shows the corresponding T_c extracted from the ROIs, where each curve can be fitted using bi-exponential decay with fitting parameters, τ_1 and τ_2, shown in Figure S21g and Figure S21h. By applying Method 2, τ_1 (red line in Figure S21g) and τ_2 (blue lines in Figure S21h) both show upward trends for Sn-Pb binary nanocrystal systems, extremely similar to the

results from Method 1 shown in Figure S21i. The results show that although the absolute values of carrier temperature may differ between models due to different ROIs, the observed trends of hot carrier cooling in Sn-Pb binary perovskites nanocrystals are not systematically affected by the inaccuracies from different models.

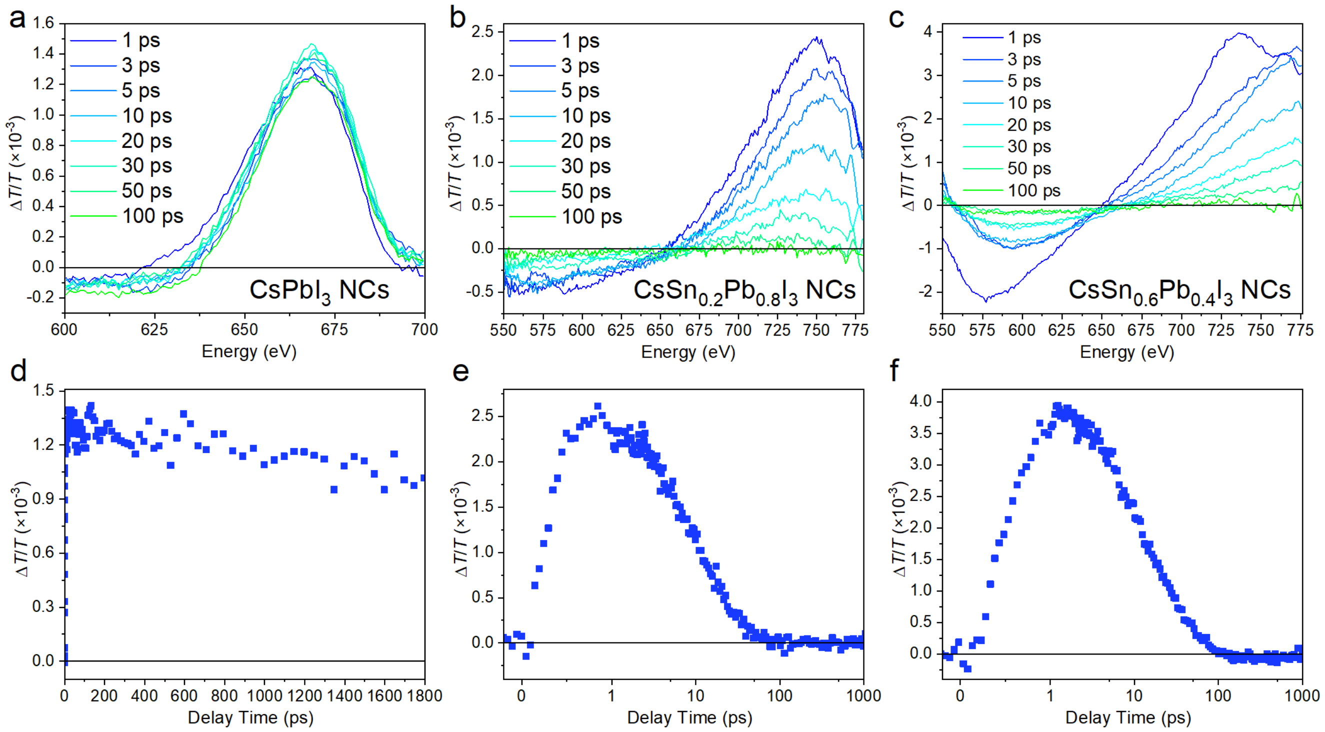

Figure S22. TA spectra and the corresponding kinetics of the ground state bleach of (a, d) $CsPbI_3$ NCs, (b, e) $CsSn_{0.2}Pb_{0.8}I_3$ NCs, and (c, f) $CsSn_{0.6}Pb_{0.4}I_3$ NCs. Even replacing a small part of Pb with Sn causes significantly enhanced charge carrier recombination rate.

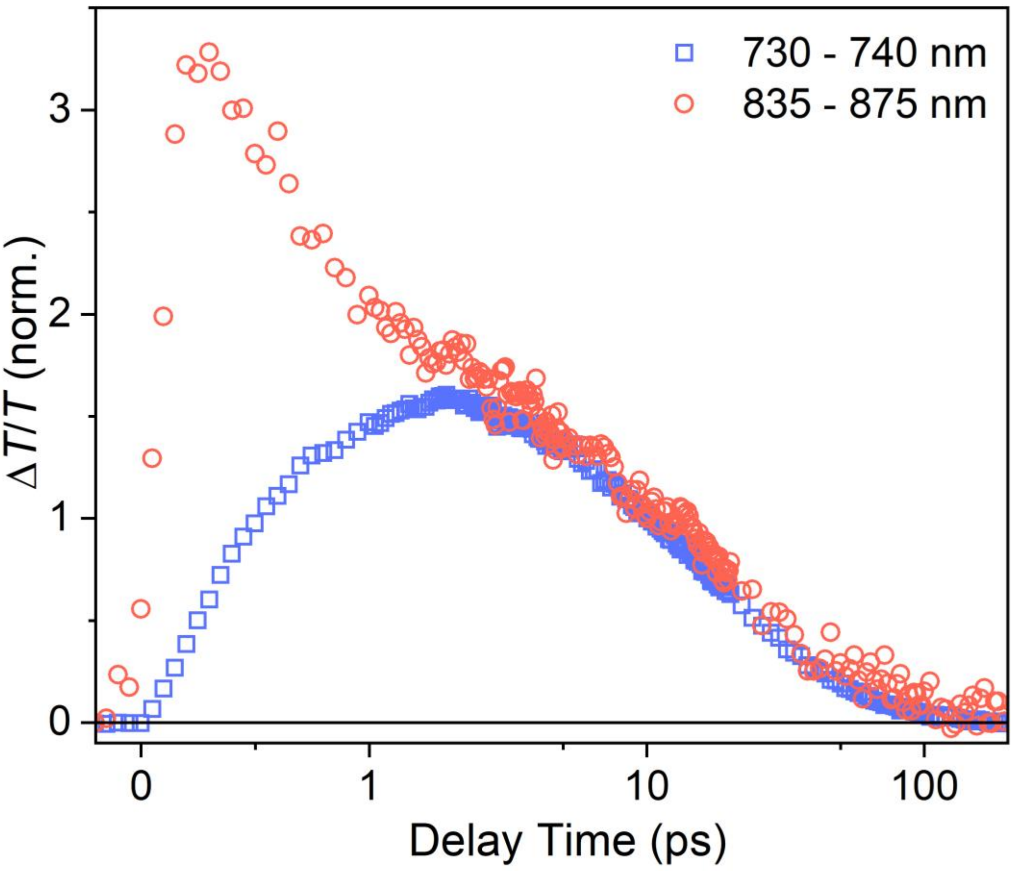

Figure S23. Normalized TA kinetics (Figure 6f, normalized at 10 ps) of the GSB and the PIA signals of Na-doped $CsSn_{0.4}Pb_{0.6}I_3$ NCs.

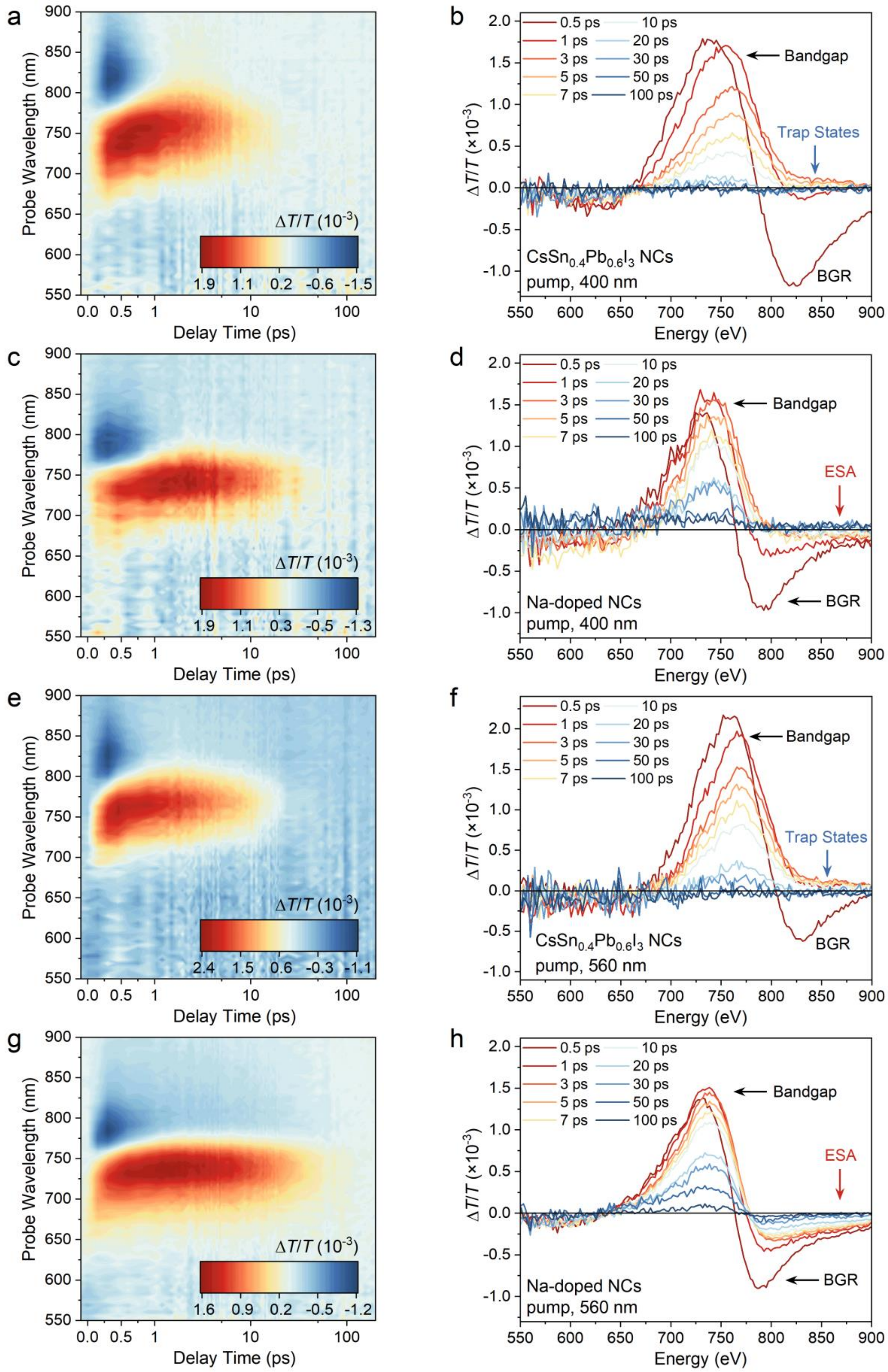

Figure S24. TA maps and TA spectra of (a, b) $CsSn_{0.4}Pb_{0.6}I_3$ NCs and (c, d) Na-doped $CsSn_{0.4}Pb_{0.6}I_3$ NCs under 400 nm pump with fluence of 1.49 μJ cm^{-2}. TA maps and TA spectra of (e, f) $CsSn_{0.4}Pb_{0.6}I_3$ NCs and (g, h) Na-doped $CsSn_{0.4}Pb_{0.6}I_3$ NCs under 560 nm pump with fluence of 1.04 μJ cm^{-2}.

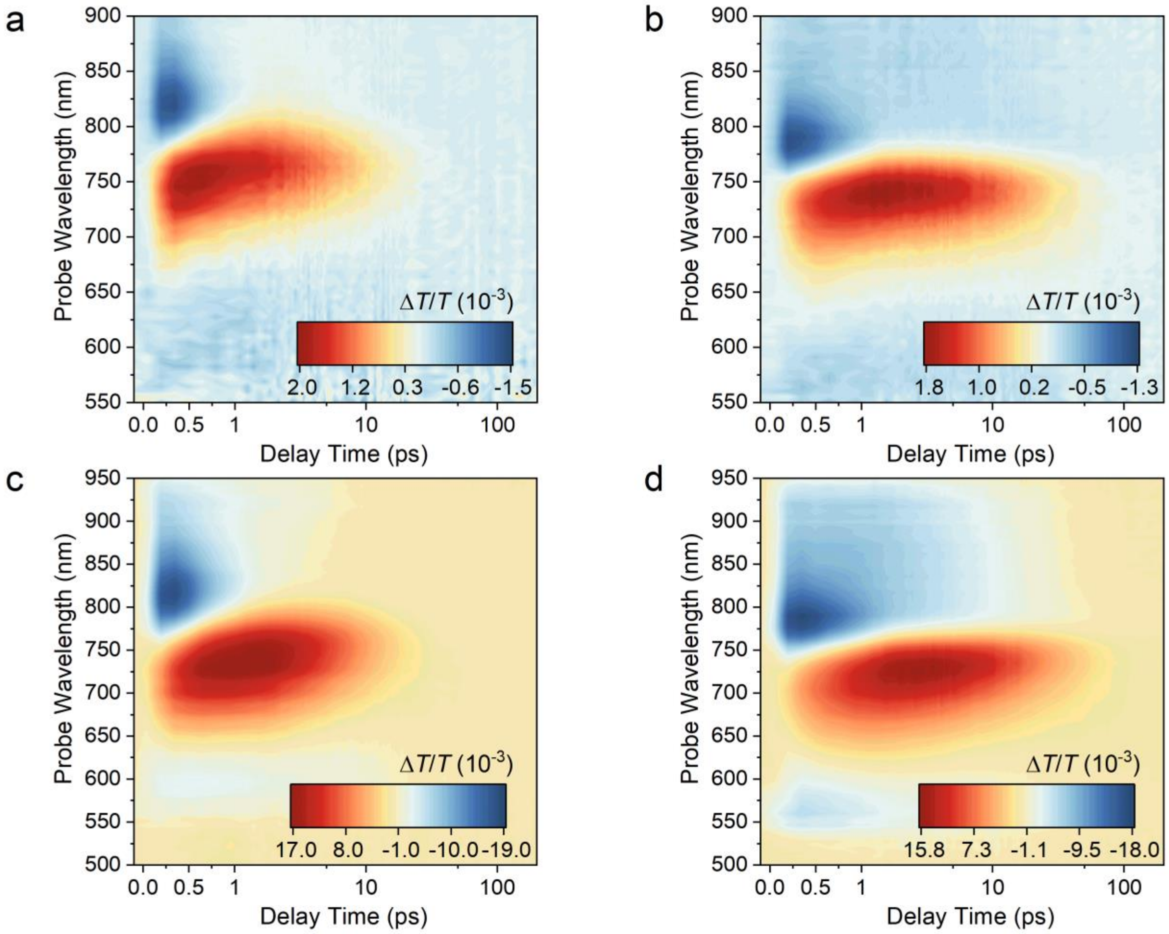

Figure S25. TA maps of $CsSn_{0.4}Pb_{0.6}I_3$ under (a) a low-fluence pump (2.70 eV, 1.5 μJ cm^{-2}) and (c) a high-fluence pump (2.70 eV, 30 μJ cm^{-2}). TA maps of Na-doped $CsSn_{0.4}Pb_{0.6}I_3$ NCs under (b) a low-fluence pump (2.70 eV, 1.5 μJ cm^{-2}) and (d) a high-fluence pump (2.70 eV, 30 μJ cm^{-2}).

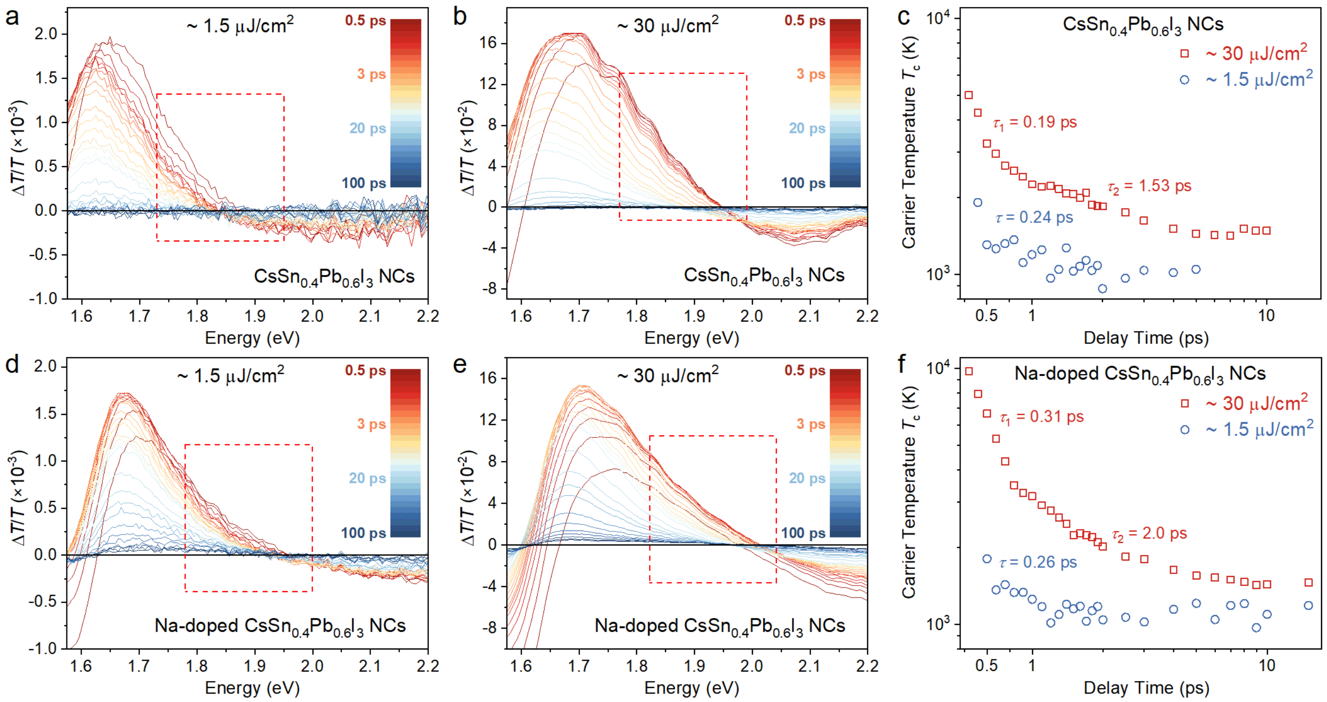

Figure 26. Hot phonon bottleneck of $CsSn_{0.4}Pb_{0.6}I_3$ NCs. Transient absorption spectroscopy of $CsSn_{0.4}Pb_{0.6}I_3$ and Na-doped $CsSn_{0.4}Pb_{0.6}I_3$ NCs under a 2.70-eV pump. **a** TA spectra under a low-fluence pump (1.5 μJ cm^{-2}), **b** TA spectra under a high-fluence pump (30 μJ cm^{-2}), and **c** extracted carrier temperature of $CsSn_{0.4}Pb_{0.6}I_3$ NCs synthesized at 170 °C. **d** TA spectra under a low-fluence pump (1.5 μJ cm^{-2}), **e** TA spectra under a high-fluence pump (30 μJ cm^{-2}), and **f** extracted carrier temperature of Na-doped $CsSn_{0.4}Pb_{0.6}I_3$ NCs synthesized at 170 °C. The red dashed boxes in (a, b, d, e) indicate the chosen area for T_c fittings.

Table S1. Fitting parameters for time-dependent carrier temperture in $MASnI_3$ NCs.

n_0 (cm^{-3})	A_1 (K)	τ_1 (ps)	A_2 (K)	τ_2 (ps)	y_0 (K)
4.08×10^{17}	2327	0.38	-	-	763
8.16×10^{17}	2905	0.42	-	-	803
1.63×10^{18}	2906	0.40	151	7.7	732
3.26×10^{18}	4085	0.37	249	7.9	772
4.89×10^{18}	7343	0.33	375	7.1	795
6.52×10^{18}	18365	0.27	835	6.4	691
8.16×10^{18}	18933	0.29	1089	6.4	722
Fitting function, $T_c(t) = A_1\exp(-(x-0.1)/\tau_1) + A_2\exp(-(x-0.1)/\tau_2) + y_0$					

References

1 Price, M. B. et al. Hot-carrier cooling and photoinduced refractive index changes in organic-inorganic lead halide perovskites. *Nat. Commun.* **6**, 8420 (2015).

2 Yang, Y. et al. Observation of a hot-phonon bottleneck in lead-iodide perovskites. *Nat. Photon.* **10**, 53–59 (2016).

3 Richter, J. M. et al. Ultrafast carrier thermalization in lead iodide perovskite probed with two-dimensional electronic spectroscopy. *Nat. Commun.* **8**, 376 (2017).

4 Shen, Q. et al. Slow hot carrier cooling in cesium lead iodide perovskites. *Appl Phys Lett* **111**, 153903 (2017).

5 Li, M. J. et al. Slow cooling and highly efficient extraction of hot carriers in colloidal perovskite nanocrystals. *Nat. Commun.* **8**, 14350 (2017).

6 Chen, J. S., Messing, M. E., Zheng, K. B. & Pullerits, T. Cation-dependent hot carrier cooling in halide perovskite nanocrystals. *J. Am. Chem. Soc.* **141**, 3532–3540 (2019).

7 Papagiorgis, P., Protesescu, L., Kovalenko, M. V., Othonos, A. & Itskos, G. Long-lived hot carriers in formamidinium lead iodide nanocrystals. *J Phys Chem C* **121**, 12434–12440 (2017).

8 Lim, J. W. M. et al. Hot carriers in halide perovskites: How hot truly? *The Journal of Physical Chemistry Letters* **11**, 2743-2750 (2020).

9 Shah, J. & Leite, R. C. C. Radiative recombination from photoexcited hot carriers in GaAs. *Phys. Rev. Lett.* **22**, 1304-1307 (1969).

10 Shah, J., Lin, C., Leheny, R. F. & DiGiovanni, A. E. Pump wavelength dependence of hot electron temperature in GaAs. *Solid State Commun* **18**, 487-489 (1976).

11 Xu, Z. Y. & Tang, C. L. Picosecond relaxation of hot carriers in highly photoexcited bulk GaAs and GaAs-AlGaAs multiple quantum wells. *Appl Phys Lett* **44**, 692-694 (1984).

12 Leo, K. & Rühle, W. W. Influence of carrier lifetime on the cooling of a hot electron-hole plasma in GaAs. *Solid State Commun* **62**, 659-662 (1987).

13 Motisuke, P., Argüello, C. A. & Leite, R. C. C. Hot electron and hot phonon contributions to radiative emission spectra in CdS at high excitation intensities. *Solid State Commun* **16**, 763-765 (1975).

14 Kash, K. & Shah, J. Carrier energy relaxation in $In_{0.53}Ga_{0.47}As$ determined from picosecond luminescence studies. *Appl Phys Lett* **45**, 401-403 (1984).

15 Savill, K. J., Klug, M. T., Milot, R. L., Snaith, H. J. & Herz, L. M. Charge-carrier cooling and polarization memory loss in formamidinium tin triiodide. *The Journal of Physical Chemistry Letters* **10**, 6038-6047 (2019).

16 Umari, P., Mosconi, E. & De Angelis, F. Relativistic GW calculations on $CH_3NH_3PbI_3$ and $CH_3NH_3SnI_3$ perovskites for solar cell applications. *Sci. Rep.* **4**, 4467 (2014).

17 He, Y. & Galli, G. Perovskites for solar thermoelectric applications: A first principle study of $CH_3NH_3AI_3$ (A = Pb and Sn). *Chem Mater* **26**, 5394-5400 (2014).
